# Enhanced TNT Vapor Detection via a Donor–Acceptor-Based Imine Cross-Conjugated Aggregation-Induced Enhanced Emission Active Porous Polymer

**DOI:** 10.1021/acsomega.5c08259

**Published:** 2025-09-25

**Authors:** Pramod C. Raichure, Annu Agarwal, Bharat Kaushik, Ajeet Singh, Inamur Rahaman Laskar

**Affiliations:** † Department of Chemistry, 29794 Birla Institute of Technology and Science, Pilani, Pilani Campus, Vidya Vihar, Pilani, Rajasthan 333031, India; ‡ Department of Chemistry, Indian Institute of Technology (IIT), Bombay, Mumbai, India, 400076

## Abstract

The development of sensitive and selective probes for the detection of nitro explosives is critical for ensuring public safety and environmental monitoring. Among various detection strategies, porous materials offer significant advantages for vapor-phase detection due to their high surface area and analyte-trapping capability. In this study, we report the design and synthesis of an electron-rich system with a donor–acceptor (D–A)-based organic porous polymer (P1), incorporating triphenylamine as the electron-donating unit and imine-conjugated sulfone (SO_2_) functionalities as electron acceptors. The resulting aggregation-induced enhanced emissive (AIEE) porous network exhibits selective fluorescence quenching in the presence of nitro explosives, particularly picric acid (PA) and 2,4,6-trinitrotoluene (TNT) in aqueous media. Notably, in the vapor phase, P1 demonstrates a strong and selective response to TNT vapors with a detection limit of 50 ppb, attributed to its higher vapor pressure compared to PA. Experimental and density functional theory (DFT) mechanistic investigations revealed distinct sensing pathways: Förster resonance energy transfer governs PA detection, while photoinduced electron transfer is responsible for TNT sensing. The high porosity of the polymer, confirmed through FESEM imaging and BET surface area analysis, facilitates efficient analyte capture, contributing to its superior vapor-phase sensitivity.

## Graphical abstract

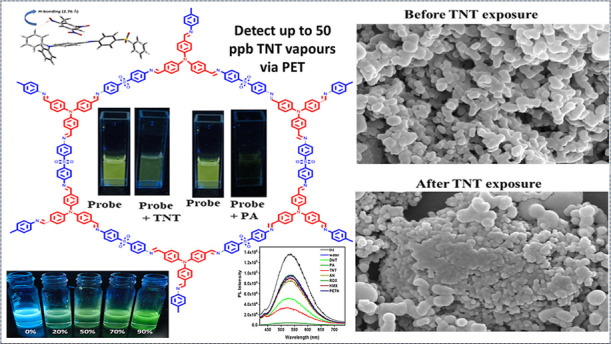

## Introduction

Luminescent materials, known for their high sensitivity, selectivity, low detection time, portability, and cost-effectiveness, are widely used in sensing.^1–5^ The detection of nitro explosives is of paramount importance for both national security and environmental safety.^6,7^ Despite their relative stability under ambient conditions, many nitroaromatic compounds can be highly sensitive to external stimuli, posing a risk of unintended detonation.^8^ Common explosives such as picric acid (PA), 2,4,6-trinitrotoluene (TNT), dinitrotoluene (DNT), 1,3,5-trinitro-1,3,5-triaza-cyclohexane (RDX), 1,3,5,7-tetraazacyclooctane (HMX), pentaerythritol tetranitrate (PETN), and ammonium nitrate (AN) are not only widely used in military and industrial applications but also are significant environmental pollutants due to their toxicity and persistence. Out of these explosive compounds, PA and TNT are most commonly used.^9,10^ TNT is a second-grade explosive that frequently contains DNT impurities, while PA sees extensive use in dye and chemical industries.^11,12^ The environmental release of these compounds contaminates water, soil, and air, necessitating detection methods that are both highly sensitive and selective, particularly in aqueous and vapor phases, where concentrations are often in the parts-per-billion (ppb) range due to low solubility and vapor pressure.^13^

Vapor-phase detection, in particular, poses a significant challenge due to the extremely low concentrations of explosive vapors under ambient and enclosed conditions. Effective vapor-phase sensors must fulfill several criteria: (1) should possess strong D–A character to enable efficient charge transfer (CT) with electron-deficient nitroaromatics. (2) Feature electronic energy levels (especially lowest unoccupied molecular orbital (LUMO)) suitably aligned with the analytes to facilitate CT mechanisms such as photoinduced electron transfer (PET) or a spectral overlap between the probe’s emission and the analyte’s absorption to incur Förster resonance energy transfer (FRET). (3) Should exhibit high porosity to enhance analyte capture.

Porous organic polymers, especially those with donor–acceptor architectures, are well suited to such applications. D–A systems enhance intramolecular charge transfer (ICT), improve photophysical responses, and enable a tailored energy level alignment. Prior reports have demonstrated the use of such systems for sensing nitroaromatics like PA, DNT, and TNT,^10,14–24^ with the best vapor-phase detection limits reaching as low as five ppb.^22^ However, improving response times and achieving consistent performance across both solution and vapor phases remain key challenges.^7,22,25^ Earlier, our group also reported one conjugated polymer for TNT vapor sensing, which utilizes triphenylamine (TPA) as a donor unit and triazine as an acceptor. The detection limit was 698 ppb for TNT vapors.^26^ A low limit of detection (LOD) in the vapor phase compared to that in the solution state is due to the less porous nature of the oligomer.

In this work, we designed a noble D–A porous polymer (P1) incorporating TPA as a strong donor connecting with the accepting unit, imine-linked SO_2_ groups as potent acceptors.^26–29^ The TPA unit was chosen for its strong donating ability and solid-state emissive behavior, which are further enhanced by incorporating chains in the three directions to induce porosity. The resulting polymer exhibited a high surface area, facilitating efficient analyte trapping, as confirmed by FESEM and BET analyses, thus resulting in a selective and sensitive detection of PA and TNT. Mechanistic studies reveal that FRET and PET dominate the detection pathways for PA and TNT, respectively. Overall, this work highlights a rational strategy to construct strong D–A character porous polymers with dual-phase (aqueous and vapor) sensing capabilities for nitroaromatic explosives.

## Experimental Section

### Synthesis

The synthesis of the polymer was done in three steps.

**First step**: In the first step, the formylation of the two phenyl rings of the TPA was done according to the reported procedure, and NMR characterization was done and shown in Supporting Information Figures S1 and S2.^30^ **Second step**: In the second step, the formylation of the third phenyl ring of the product obtained from the first step was done according to the reported literature, and NMR characterization was done and shown in Supporting Information Figures S3 and S4.^31^ **Third step**: The products obtained from steps 2 (M1) and 4,4′-sulphonyldianiline (M2) were dissolved in tetrahydrofuran (THF) solvent in a two-neck round-bottom flask. A catalytic amount of acetic acid was added, and the reaction mixture was stirred for 48 h at room temperature. After completion of the reaction, a dichloromethane (DCM) water workup of the reaction mass was done, and the organic layer was collected and reduced using a rotary evaporator after drying over MgSO_4_. A large amount of methanol was used to distribute the reaction mixture. Several times, centrifugation and washing led to product polymer P1.

### Computational Study

Density functional theory (DFT) calculations on a monomeric unit of the polymer were performed on Gaussian 16 software. cam-B3LYP was utilized as a functional, as it is better suited for donor–acceptor systems, and 6–31+G­(d,p) was used as the basis set. Highest occupied molecular orbital (HOMO) and LUMO energies were calculated in eV.

### Spectroscopic Studies

To record UV spectra, 2 mg of the compound was dissolved in 5 mL of THF solvent. The same solution of P1 was utilized to record the emission spectra. Solvatochromic studies were performed, and the Lippert-Mataga plot was plotted. Details were provided in the Supporting Information.

An aggregation-induced emission property study of the compound was done in THF and water solvent systems. First, a stock solution of probe in THF (i.e., 2 mg of P1 in 10 mL THF) was prepared, and then 0.2 mL of the stock solution was transferred to each 5 mL glass vials. Then, 0, 20, 50, 70, and 90% water fraction solutions were prepared by adding the required amounts of THF and water in each vial.

### Nitro-Explosive Detection Studies

#### In the Solution Phase

A 90% water: THF AIEE solution was used for detection studies of the probe toward PA and 2,4,6-trinitrotoluene (TNT). First of all, a selectivity study was done in a 10^–3^ M aqueous solution of nitro explosives, including DNT, PA, TNT, AN, RDX, HMX, and PETN. After that, titration of the probe with 10^–3^ M PA and TNT was done to calculate the Stern–Volmer (S–V) quenching constant (*K*_SV_) and detection limit.

### In the Vapor Phase

For vapor phase detection of TNT, Whatman 1 filter paper strips were dipped into the THF solution of the probe and dried well. The saturated vapors of TNT were exposed to the paper strips, and PL spectra were recorded (experimental setup is shown in Supporting Information Figure S20). To check the stability of the filter paper strips, blank readings were taken after two min intervals (Supporting Information Figure S18).

### Quenching Constant Calculation

The value of the quenching constant for static quenching can be obtained by using the formula given below

$$F_{0}/F=1+K_{sv}\left[Q\right]$$

where *F*_0_ is the probe’s initial intensity without the addition of analyte, *F* is the intensity of the probe after adding the quencher’s (PA and TNT) known concentration, *K*_sv_ is the S–V quenching constant, and [*Q*] is the concentration of the quencher (PA and TNT).

The value of the apparent quenching constant (*K*_app_), where both static and dynamic quenching were present, can be calculated using the formula

$$\frac{F_{0}}{F}=1+k_{app}\left[Q\right]$$

where

$$k_{app}=\left[\frac{F_{0}}{F}-1\right]\frac{1}{\left[Q\right]}=\left(K_{D}K_{s}\right)+K_{D}K_{S}\left[Q\right]$$

here, *K*_D_ is the dynamic quenching constant and *K*_S_ is the static quenching constant.

### Detection Limit Calculation

The Stern–Volmer plot was plotted after titrating a 90% water fraction of THF AIEE solution of the probe with different concentrations of PA and TNT , which was used to determine the detection limit for the probe. The plot’s straight-line portion was used to calculate the slope (*k*) value. To obtain a signal-to-noise ratio, 20 PL spectra were recorded with the slit width set to the lowest possible value. The standard deviation (σ) for these spectra was then computed.

The LOD was derived by entering the obtained values of *k* and σ into the equation below.

LOD=3σ/k

### Förster Resonance Energy Transfer Calculations

The overlap integral between polymer emission and PA absorption was calculated. Emission spectra were recorded using a 90% water: THF AIEE solution of the polymer, and a 10^–5^ M aqueous PA solution was used for the absorption spectra of PA. The value of the overlap integral 9.41 × 10^14^ was calculated by using the formula below.

$$J\left(\lambda \right)=\frac{\int_{0}^{\infty }F_{D}\left(\lambda \right)\varepsilon_{A}\lambda^{4}d\lambda }{\int_{0}^{\infty }F_{D}\left(\lambda \right)d\lambda }$$

where *J* is the overlap integral, *F*_D_ is the fluorescence intensity of the donor in the absence of an acceptor, and ε_A_ is the extinction coefficient of the acceptor.

The value of *R*_0_ was calculated using the formula below and found to be 3.26 nm, which is in the accepted range 1–10 nm.

$$R_{0}^{6}=8.79\times 10^{-5}\left(K^{2}\eta^{4}\varphi_{D}J\left(\lambda \right)\right){Å}^{6}$$

where *K* is the orientation of the donor and acceptor in the excited state, which is considered as 2/3, η is the refractive index of the medium,^32^ φ_D_ is the quantum yield of the donor, which is 8.38%, and *J*(λ) is the overlap integral.

The efficiency of the energy transfer (*E*) was calculated from the formula given below.

$$E=1-\frac{F_{DA}}{F_{D}}$$

where *F*_DA_ is the fluorescence intensity of the probe in the presence of the acceptor (6536) and *F*_D_ is the fluorescence intensity of the probe in the absence of the acceptor (134550). The calculated efficiency of energy transfer comes out to be 95.15%.

Using the values of *E* and *R*_0_, we can calculate the value of r as 1.968 nm according to the equation.

$$E=\frac{R_{0}^{6}}{R_{0}^{6}+r^{6}}$$

The value of the rate of energy transfer was calculated using the equation given below, and the value obtained is 4.03 × 10^10^ s^–1^.

$$k_{t}\left(r\right)=\frac{1}{\tau_{D}}{\left(\frac{R_{0}}{r}\right)}^{6}$$

where τ_D_ is the donor’s lifetime in the excited state in the absence of the acceptor, which is 0.5 ns.

## Results and Discussion

### Synthesis and Characterization

The synthesis of the polymeric compound was done in three steps (Experimental Section). In brief, the formylation of a two-phenyl ring of TPA occurred in the first step. NMR spectra of the same are shown in Supporting Information Figures S1 and S2. In the second step, 4,4′,4″-formyl-triphenylamine was synthesized and characterized (Supporting Information Figure S3 and S4) (Scheme 1). In the third step, the polymerization reaction between M1 and M2 takes place in THF solvent in the presence of a catalytic amount of acetic acid (Scheme 2). The reaction was stopped after 48 h, followed by the reaction mixture being extracted using DCM and water workup. It was purified by precipitation from DCM into the bulk of methanol several times, followed by centrifugation.^33^ A polymer P1 as a product was obtained. NMR (Supporting Information Figures S5, S6) spectra were recorded to confirm the formation of the polymer. The molecular weight (*M̅*_w_) of the polymer P1 was observed at ∼44000 Da with a PDI of 1.26 from GPC analysis (Supporting Information Figure S7). The expected polymeric structure of the polymer is shown in Scheme 3.

**Scheme 1:**
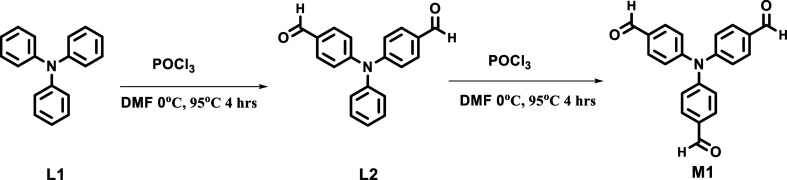
Synthetic Route for the Synthesis of Monomer (M1)

**Scheme 2:**
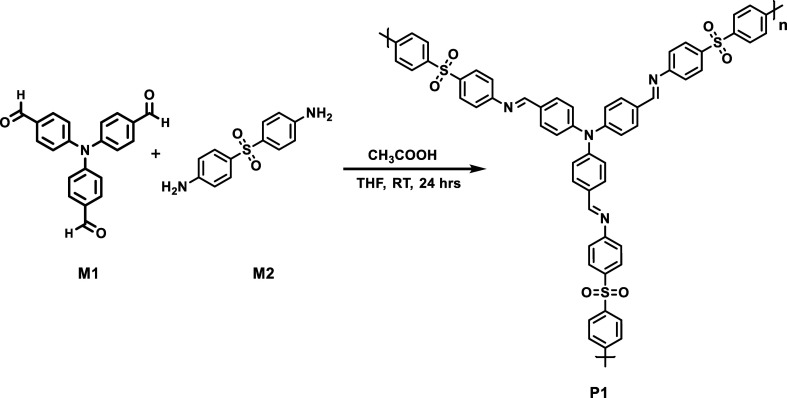
Synthetic Route for (P1) Polymer Synthesis

**Scheme 3:**
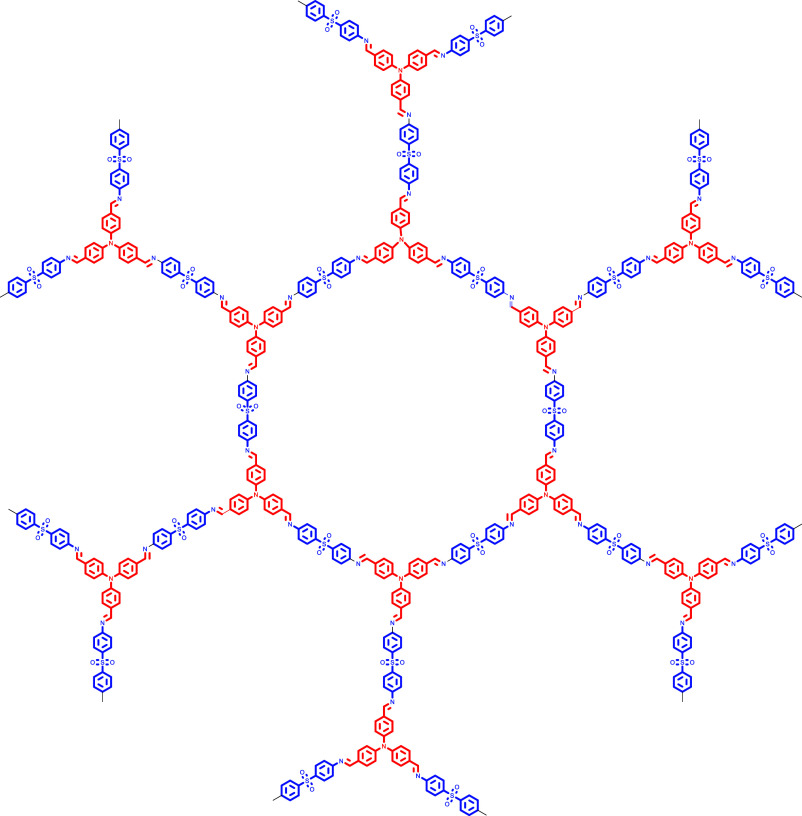
Expected Network of the Synthesized Polymer

### Photophysical Property Studies

The absorption and emission spectra of the polymer were recorded in UV-grade THF (Figure 1a). The absorption spectrum exhibited multiple peaks with a notable band around 380 nm. This band was attributed to have a CT character, based on our natural transition orbital (NTO) analysis (vide infra, Figure 3c). Additional peaks observed in the 250 nm to 320 nm range correspond to π → π* transitions, consistent with literature reports on similar molecular systems.^34,35^

**Figure 1:**
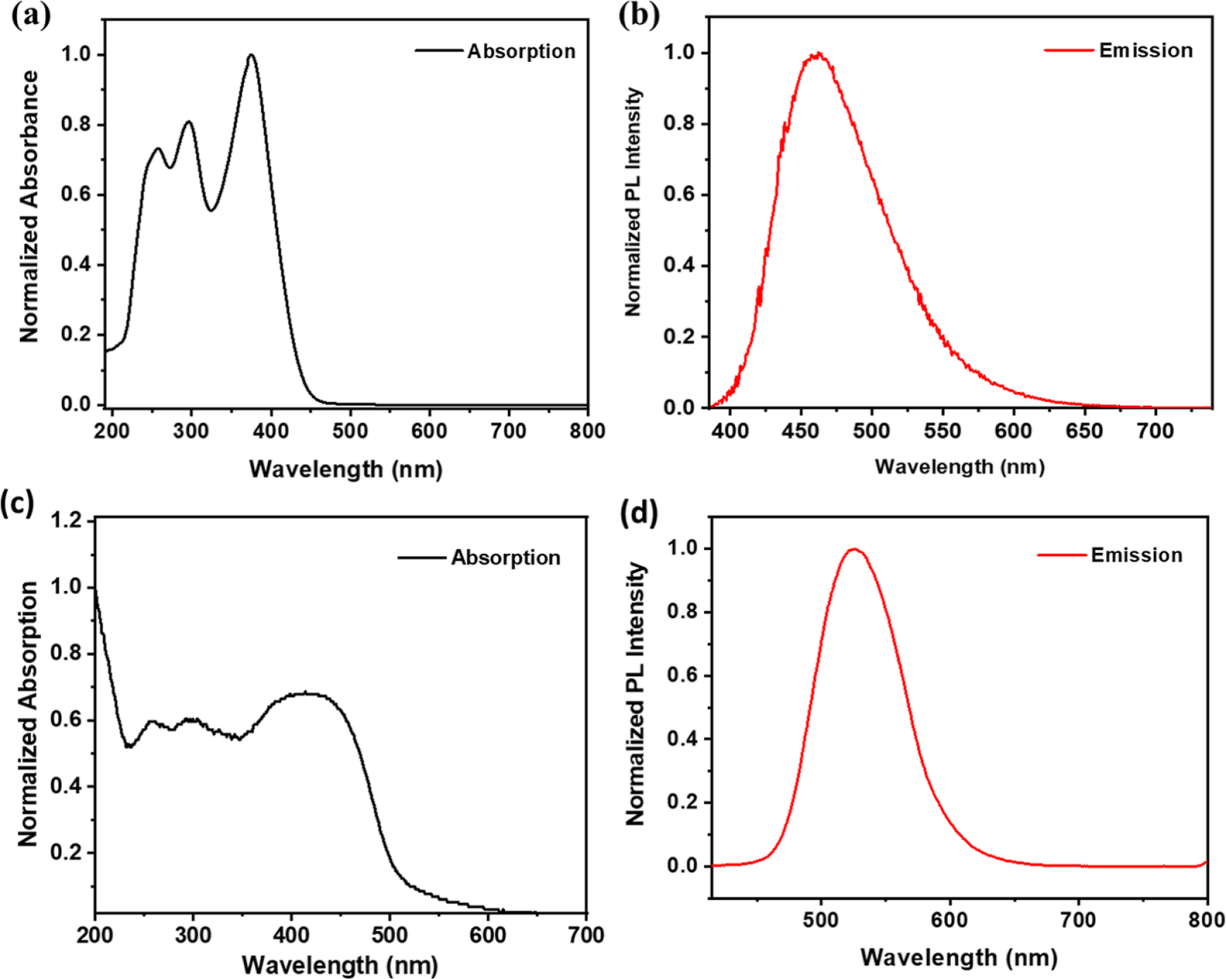
(a,b) Absorption and emission spectra of the polymer recorded in UV grade THF solvent (λ_ex_ = 380 nm), respectively, (c,d) absorption and emission spectra of the polymer recorded in solid state (λ_ex_ = 416 nm), respectively.

Upon excitation at 380 nm, the polymer displayed a strong photoluminescence (PL) with an emission maximum at around 460 nm, as shown in Figure 1b. In the solid state, the absorption spectrum featured a broad band centered at 422 nm with a tail extending up to 600 nm, while the emission spectrum was red-shifted, peaking at 526 nm (Figure 1c, d). This red shift, along with a significant enhancement in emission intensity, is likely due to reduced nonradiative decay pathways upon molecular rigidification in the solid state. The rigidification reduces the degrees of freedom of nonradiative decay by minimizing the rotations and vibrations.^36,37^ Excitation-dependent emission spectra of the polymer in the solid as well as in the solution state have been shown in Supporting Information Figure S8.

### Solvatochromic Study

The monomer unit in polymer P1 exhibits D–A characteristics, making its photophysical properties sensitive to solvent polarity.^38^ DFT findings also suggested S_1_ to have CT-type character. To investigate this, the absorption and emission spectra of P1 were recorded in solvents of varying polarity, including toluene, THF, DCM, acetone, *N*,*N*-dimethylformamide (DMF), and *N*,*N*-dimethyl sulfoxide (DMSO). Physical properties of the solvents are summarized in Supporting Information Table S1. The UV–vis absorption spectra showed minimal changes across the solvent series, indicating a limited influence of solvent polarity on the ground-state electronic transitions. In contrast, the emission spectra displayed a pronounced red shift, with the emission maximum shifting from 427 nm in toluene to 482 nm in DMSO, as shown in Figure 2. This significant solvatochromic behavior suggests a strong CT character of the S_1_ state. Thus, it establishes the robustness of the DFT results. A Lippert–Mataga plot for the polymer was plotted, which correlates orientation polarizability with Stokes shift (Supporting Information Figure S9). A positive slope was observed, which indicates a strong ICT character in the polymer.^39^ Apart from that, Dimroth–Reichardt polarity parameter *E*_T_(30) was plotted against the absorption (*E*_a_) and emission energy (*E*_f_) (Supporting Information Figure S10­(a)). A negative slope indicates ICT character, and a more negative slope of *E*_f_ compared to *E*_a_ implies the excited state is more affected by solvent polarity than the ground state. Additionally, the Dimroth–Reichardt polarity parameter *E*_T_(30) was plotted against the Stokes shift (Supporting Information Figure S10­(b)). A positive slope indicates the CT mechanism is ICT. Entries for the same are provided in Supporting Information Table S2.

**Figure 2:**
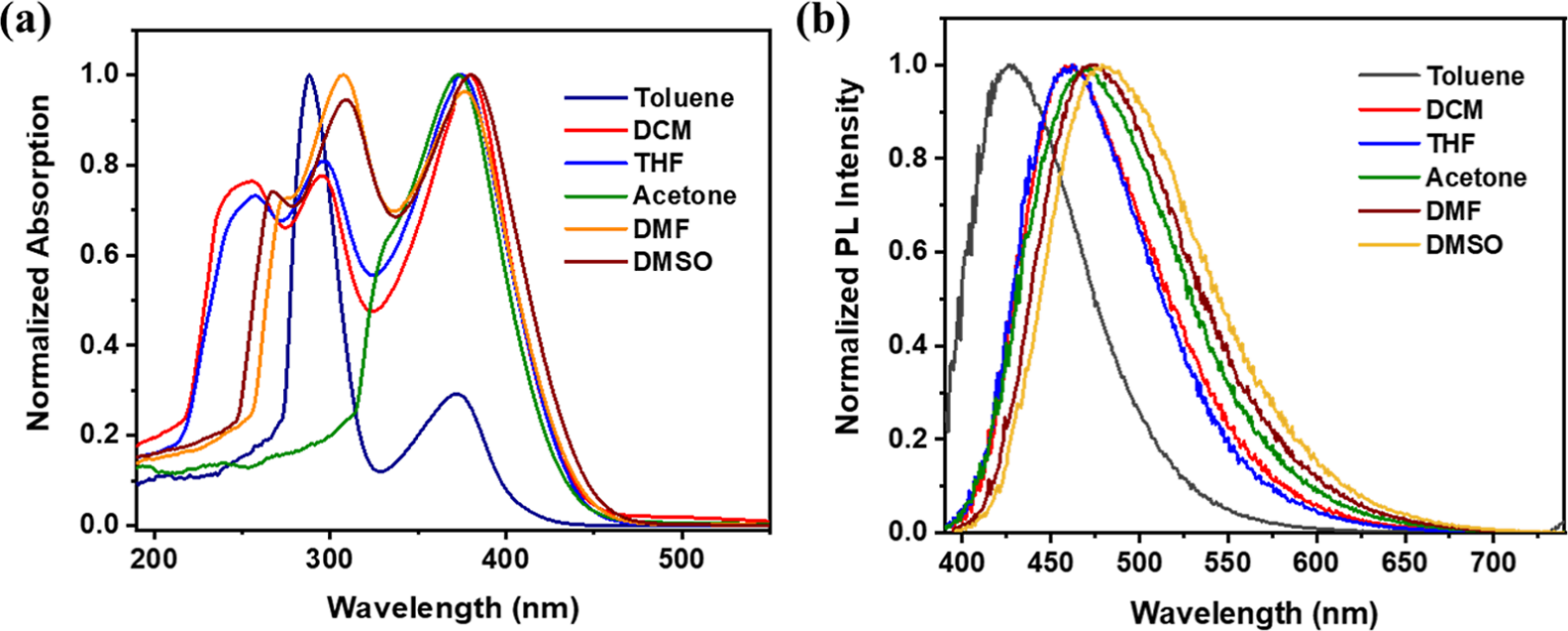
(a) Absorption spectra and (b) emission spectra of the polymer P1 taken in various solvents (Toluene, DCM, THF, Acetone, DMF, and DMSO) (λ_ex_ = 370—380 nm).

### Computational Studies

DFT calculations were carried out to study the monomeric unit using the Gaussian 16 software package.^40,41^ Geometry optimization was performed using the LRC hybrid functional cam-B3LYP, which effectively accounts for noncovalent interactions.^42^ The 6–31 + G (d,p) basis set was employed to incorporate both diffuse and polarization functions (Figure 3a).^43^

**Figure 3:**
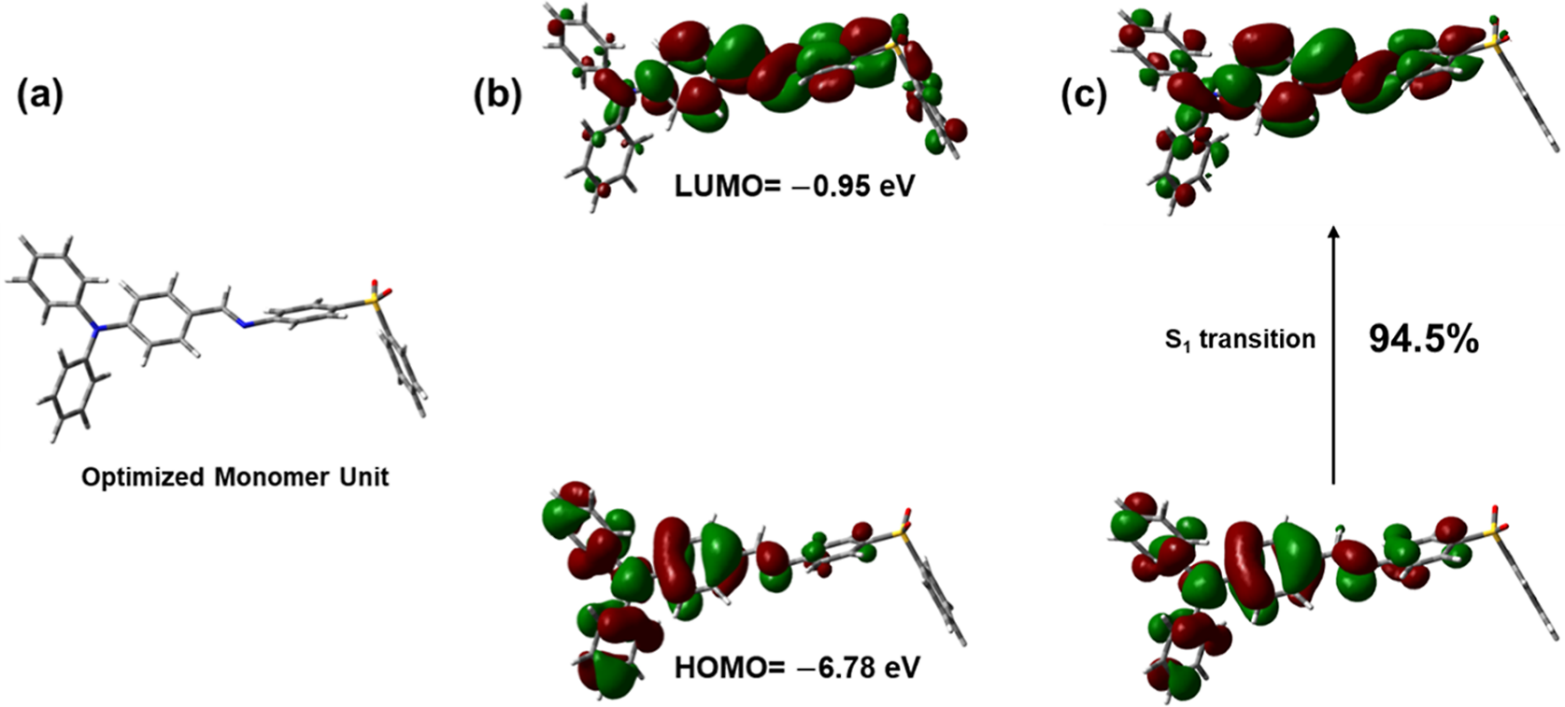
(a) Optimized structure of the monomer unit and (b) its corresponding HOMO and LUMO using cam-B3LYP long-range corrected (LRC) functional and 6-31G+(d,p) basis set (c) NTOs of S_1_ transition and its occupancy.

The calculated energies of the HOMO and the LUMO were −6.78 eV and −0.95 eV, respectively. As shown in Figure 3b, the HOMO is primarily delocalized over the TPA moiety, whereas the LUMO is mainly localized on the imine and adjacent phenyl ring.

To gain insight into the excited-state properties, time-dependent DFT calculations were performed. The first singlet excited state (S_1_) was predicted at 338 nm (3.66 eV) with a significant oscillator strength (*f* = 1.20), indicating a strongly allowed transition. The other five calculated electronic transitions were excluded from further analysis due to their significantly lower oscillator strengths. The molecular orbitals involved in the S_1_ transition and their corresponding contributions are summarized in Table 1, highlighting the contributions of different orbitals in the transition. To further clarify the nature of the electronic transition, an NTO analysis was performed. The NTOs corresponding to the S_1_ state are shown in Figure 3c. The spatial distributions of the particle and hole closely resemble those of LUMO and HOMO, respectively. This analysis confirms that the S_1_ transition is characterized by substantial CT from the TPA donor unit to the imine acceptor, with minor contributions from local excitations primarily localized on the TPA ring.

**Table 1:** Orbitals Involved in the S_1_ Transition and Their Respective Percentage Contributions

orbitals involved	% contribution
HOMO-1 → LUMO	5.8
HOMO → LUMO	79.6
HOMO → LUMO + 1	6.8

### Aggregation-Induced Enhanced Emission in a THF and Water Mixture

To assess the compatibility of the probe in aqueous media, an essential requirement for nitro-explosive sensing, PL spectra were recorded in THF–water mixtures. The emission intensity of P1 decreased sharply upon increasing the water content from 0% to 20%, as shown in Figure 4. This quenching is attributed to the increased polarity of the solvent mixture, which promotes nonradiative decay processes.^44^

**Figure 4:**
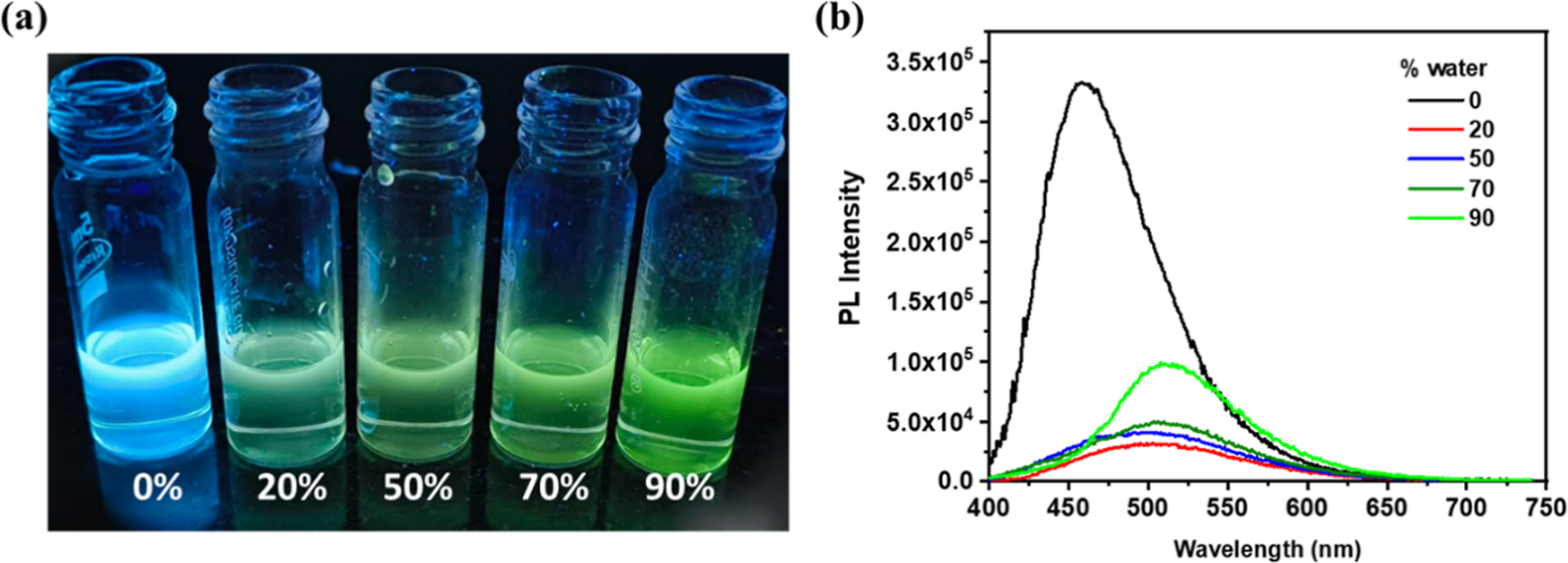
(a) Images of THF solution of the probe with an increasing water fraction under a UV lamp (λ_ex_ = 365 nm); (b) PL spectra of the water–THF study of the polymer excited at 380 nm.

Interestingly, with a further increase in the water fraction beyond 20%, a gradual enhancement in emission intensity was observed. This behavior is likely due to the formation of molecular aggregates in higher water content, which restricts intramolecular motions and reduces nonradiative decay pathways through enhanced rigidification. A progressive increase in aggregate size with water content is expected,^45^ as supported by the DLS study, contributing to the observed rise in PL intensity (Supporting Information Figure S11­(c,d)).

To further confirm that emission enhancement originates from reduced nonradiative decay due to restricted molecular motion or reduced degrees of freedom in vibrations and rotations,^46^ a similar experiment was conducted using THF-poly­(ethylene glycol) (PEG 400) mixtures. PEG, being a highly viscous solvent, imposes conformational constraints on molecular rotation and vibration (i.e., degrees of freedom). The PL intensity initially decreased up to 50% PEG fraction, likely due to the polarity effect, but increased at higher PEG concentrations, as shown in Supporting Information Figure S11­(a). This indicates that at lower PEG content, solvent polarity dominates, while at a higher PEG content, the rigidity leads to suppression of nonradiative decay. In the above case, a red shift in emission was also observed, attributed to increased solvent polarity at the probe-water interface.^47–49^ Time-resolved PL measurements of the probe in a 90% water–THF mixture AIEE solution revealed a short lifetime of 0.5 ns (Supporting Information Figure S12), with a quantum yield of 8.8%.

### Sensing of Nitro Explosives in a THF and Water Mixture

TPA-based luminophores, due to their electron-rich character, are well-suited for detecting electron-deficient nitroaromatic compounds.^50,51^ Accordingly, the sensing capability of polymer P1 was evaluated against a series of nitro-explosive analytes. The selectivity study was conducted using 10^–3^ M aqueous solutions of various nitro compounds, including DNT, PA, 2,4,6-trinitrotoluene (TNT), AN, 1,3,5-trinitro-1,3,5-triaza-cyclohexane (RDX), 1,3,5,7-tetranitro-1,3,5,7-tetraazacyclooctane (HMX), and PETN. For each measurement, 200 μL of the respective analyte solution was added to a 90% water–THF AIEE solution of the probe, and the resulting PL spectra were recorded (Figure 5).

**Figure 5:**
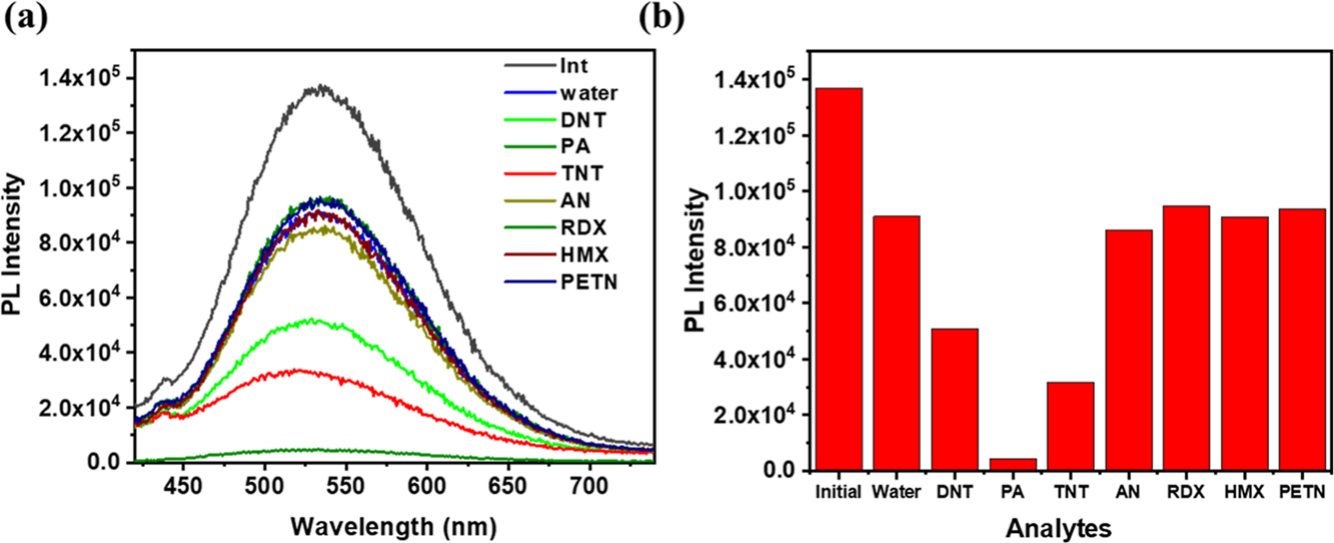
Selectivity study of the 90% water: THF AIEE solution of the polymer toward nitro explosives. (a) PL spectra of the selectivity study (λ_ex_ = 380 nm), and (b) bar graph of the PL intensity plot.

Among the tested analytes, only PA and TNT caused a significant quenching of the probe’s emission intensity, indicating a strong interaction and effective sensing. The other compounds showed a negligible effect on the luminescence signal. Based on this selectivity, the LOD for PA and TNT was subsequently calculated.

### Picric Acid Detection in the Aqueous Phase

A significant decrease in PL intensity was observed upon the addition of an aqueous solution of PA to the 90% water-THF AIEE solution of the probe. To investigate this interaction in detail, a PL titration was conducted using a 10^–3^ M aqueous PA solution (Figure 6a). The corresponding S–V plot (Figure 6b) exhibited an upward curvature, suggesting the involvement of both static and dynamic quenching mechanisms.^52^ From the straight line fit of the S–V plot, the slope was determined to be 0.031, and the apparent association constant (*K*_app_) was calculated as 8.37 × 10^4^ M^–1^.^53^ The linear fit is shown in Supporting Information Figure S13a. Furthermore, the LOD for PA was estimated to be 1.5 ppm, demonstrating the probe’s high sensitivity toward PA. All of the calculations are shown in the Supporting Information.

**Figure 6:**
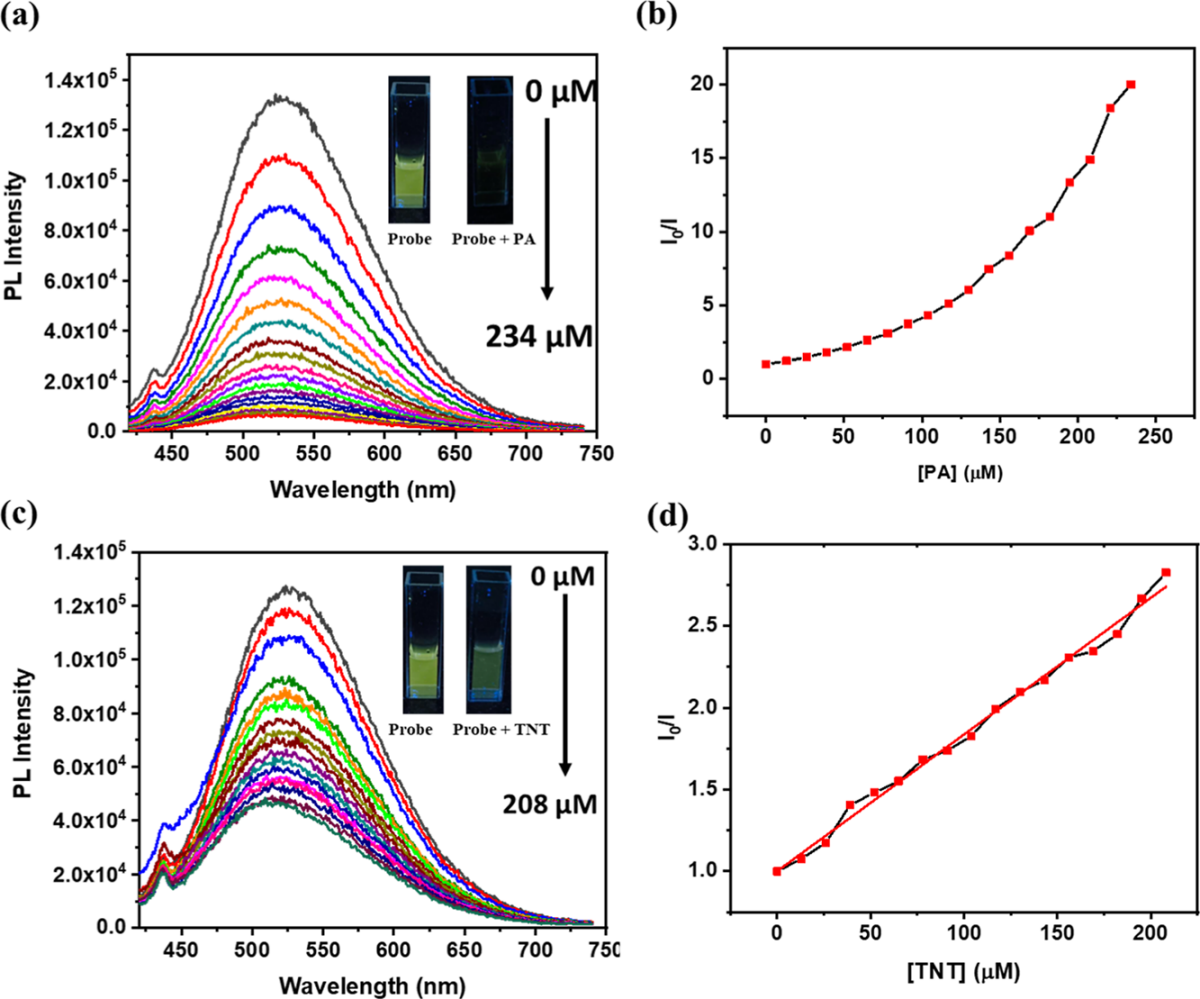
Sensitivity study of the probe toward PA and TNT. PL titration plot of the probe after addition of the analyte (λ_ex_ = 380 nm) (a) PA and (c) TNT, Stern–Volmer plot of the PL titration of the sensitivity study (b) PA and (d) TNT.

### TNT Detection in the Aqueous Phase

Following the observed selectivity of the probe toward both PA and TNT, a PL titration was also performed with a 10^–3^ M aqueous TNT solution. The 90% water–THF AIEE solution of the probe was prepared and titrated with TNT (Figure 6c). The S–V plot displayed a near-linear trend (Figure 6d), indicating that a single type of quenching, primarily static or dynamic quenching, is dominant. The quenching constant (*K*_sv_) was calculated to be 0.81 × 10^4^ M^–1^, and the LOD for TNT was determined as 5.54 ppm based on the slope of the S–V plot. The linear fit is shown in Supporting Information Figure S13­(b).

To further verify the quenching type, time-resolved PL measurements of the AIEE solution of the probe were performed in the presence and absence of TNT. Only a slight change in lifetime was observed, from 0.50 ± 0.2 ns to 0.47 ± 0.2 ns (Supporting Information Figure S14), supporting the conclusion that static quenching is the primary process.

### Filter Paper-Based Detection of PA and TNT

Encouraged by the solution-phase detection results, we further tested the probe for solid-state detection using filter paper strips. The Whatman filter paper strips were immersed in a DCM solution of the probe and then dried in a vacuum oven. Various aqueous analyte solutions, ranging from 10^–3^ M to as low as 10^–13^ M for PA and 10^–11^ M for TNT, were prepared.

For testing, 5 μL of each analyte solution was dropped onto the probe-impregnated strips, and emission changes were observed under a UV lamp (λ_ex_ = 365 nm) (Supporting Information Figure S15). The strips exhibited fluorescence quenching in the presence of PA down to 10^–11^ M and for TNT down to 10^–9^ M, demonstrating excellent sensitivity in contact mode.

## Vapor Phase Detection of TNT

Given the strong selectivity of the probe toward PA and TNT in solution, further experiments were carried out to evaluate its detection performance in the vapor phase. Probe-impregnated filter paper strips were exposed to the saturated vapors of various nitroaromatic compounds.^26,54,55^ The selectivity study (Supporting Information Figure S16) revealed that a detectable PL quenching response occurred only in the presence of TNT vapors. This selective response is attributed to TNT’s relatively high vapor pressure, which facilitates effective interaction with the probe. Other analytes did not induce any significant emission quenching under identical conditions.

To quantify the vapor-phase sensing, PL titration was conducted using probe-impregnated filter paper strips exposed to saturated TNT vapor. The corresponding S–V plot (Figure 7) exhibited an approximately linear relationship, with a slope of 0.0028. The linear fit is shown in Supporting Information Figure S13­(c). Based on this, the LOD was calculated to be 50 ppb, indicating high sensitivity. The S–V quenching constant, *K*_SV_, for TNT vapor was determined to be 6.5 × 10^5^ M^–1^, further supporting the probe’s effectiveness in vapor-phase detection.

**Figure 7:**
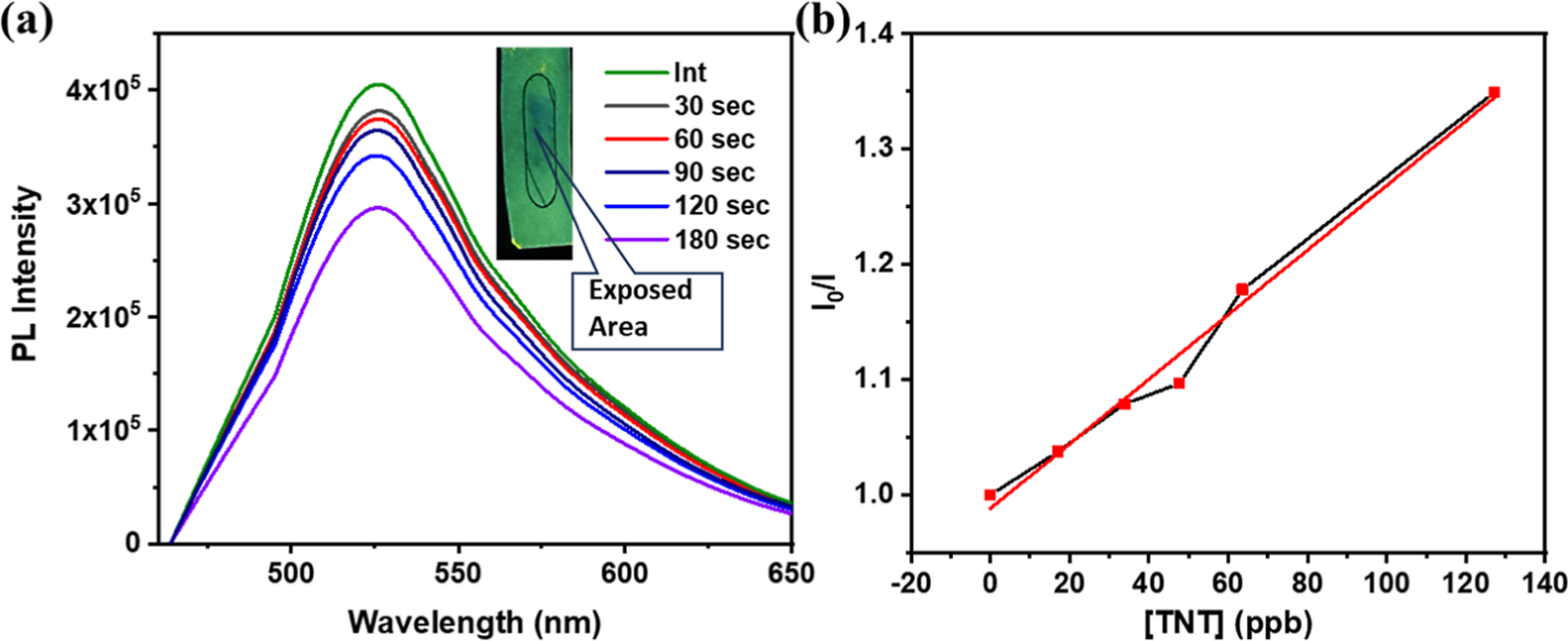
(a) PL titration plot of the sensitivity study of probe-impregnated filter paper strips (λ_ex_ = 380 nm) in the vapor phase (Int is the initial reading) (inset: image of paper strip for TNT detection). (b) Stern–Volmer plot of the PL titration, where *I*_0_ and *I* represent PL intensity of the probe in the absence and presence of the analyte, respectively.

### Mechanism of Quenching

After the type of quenching involved was established, further studies were conducted to understand the underlying mechanism. Two commonly proposed pathways for luminescence quenching of electron-rich donor systems by electron-deficient acceptors are (1) PET and (2) FRET.

In the case of PA, PET was majorly ruled out as the PA’s (dissociated form) LUMO (+0.49 eV)^56,57^ does not lie between the HOMO (−4.55 eV) and LUMO (−1.71 eV) of the probe, making electron transfer energetically unfavorable as shown in Figure 8c.^58^ To evaluate the possibility of FRET, the absorption spectrum of an aqueous PA solution was recorded and compared to the emission spectrum of the probe. A moderate spectral overlap was observed (Figure 8a), suggesting the feasibility of FRET between the donor (probe) and acceptor (PA).

**Figure 8:**
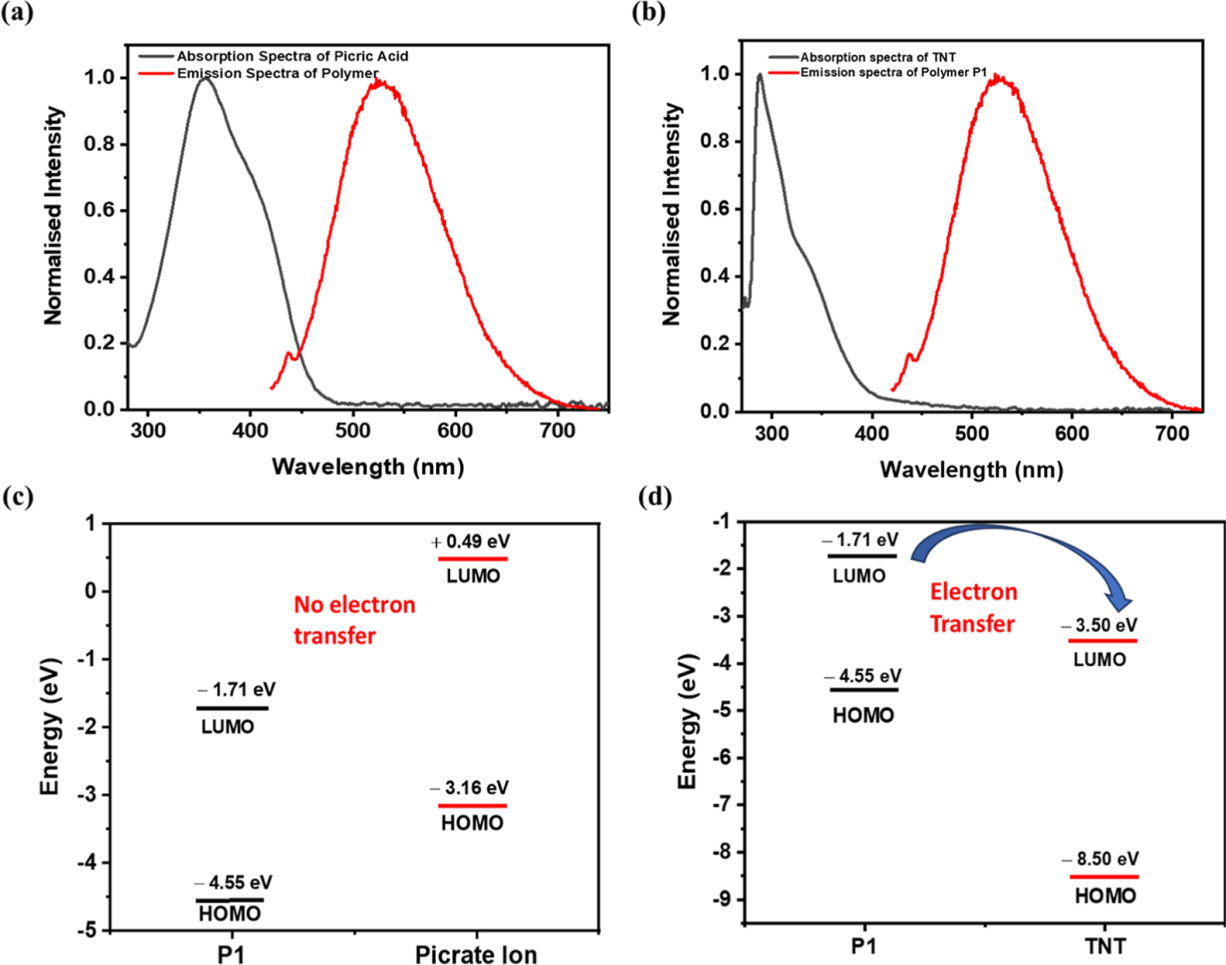
Overlap of absorption spectra of analytes (a) PA and (b) TNT and emission spectra of the polymer and (c,d) energy-level diagram representing HOMO and LUMO of probe and analyte showing the possibility of electron transfer from the probe to the analyte.

The spectral overlap integral was 9.41 × 10^14^ M^–1^ cm^–1^ nm,^4^ and the Förster distance (*R*_0_) was determined as 3.26 nm, which is an acceptable value for efficient energy transfer. The calculated FRET quenching efficiency was 95.15% (calculations are shown in the Experimental Section), confirming that FRET is the dominant quenching mechanism in the case of PA.

In contrast, for TNT, minimal spectral overlap was found between its absorption spectrum and the probe’s emission (Figure 8b), minimizing the possibility of FRET as the major mechanism. However, the energy level alignment showed a small energy gap (1.79 eV) between the LUMO of the probe (also determined experimentally, Supporting Information Figure S17) and TNT, which supports the occurrence of possible PET as the primary quenching mechanism (Figure 8d).

To support our experimental observations on TNT sensing, we performed DFT calculations. The probe was optimized by positioning TNT in a face-to-face orientation with TPA, since the HOMO was localized on it. The resulting optimized geometry revealed the presence of a hydrogen bond between the hydrogen atom of TPA and the nitro group of TNT, as illustrated in Figure 9. Frontier molecular orbital analysis showed that HOMO remained localized on the TPA moiety, while LUMO shifted entirely to TNT. This electronic distribution supports a PET mechanism as the dominant sensing pathway. Hydrogen bonding was also seen in the molecules between the NO_2_ group of TNT and one of the phenyl rings of TPA, as shown in Figure 9a. The distance between the oxygen (of TNT) and hydrogen (of TPA) was 2.76 Å. In this process, excitation might promotes an electron from the HOMO on TPA to the LUMO on TNT. This is facilitated by the possible H-bonding between the probe and analyte, followed by nonradiative relaxation, consistent with the quenching behavior observed experimentally.

**Figure 9:**
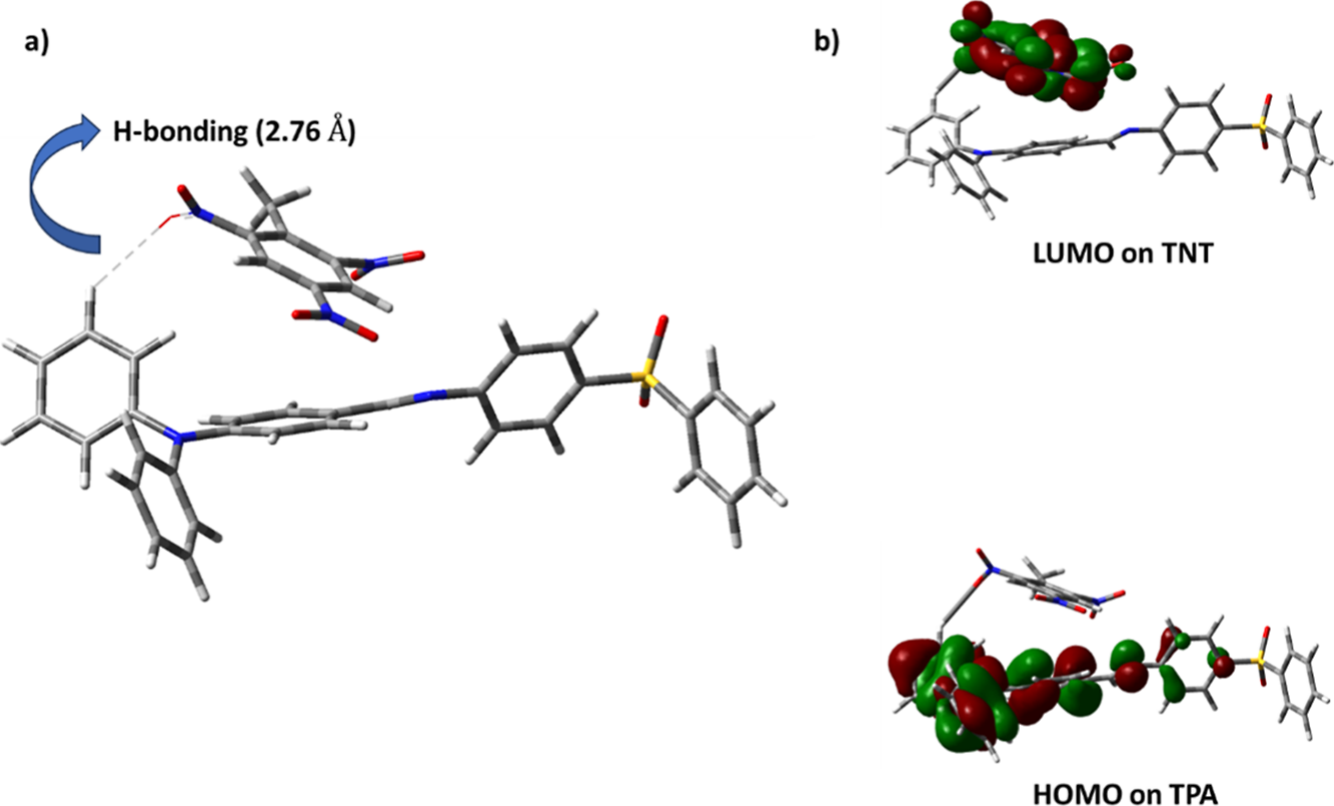
(a) Optimized structure of the probe with TNT showing hydrogen bonding and (b) corresponding HOMO–LUMO distribution.

### Morphological Analysis and Its Role in Vapor-Phase Sensitivity

To further quantify the porosity, nitrogen gas adsorption–desorption experiments were conducted at 77 K, and a Brunauer–Emmett–Teller (BET) analysis was performed (Supporting Information Figure S19). The resulting isotherm exhibited a H3-type hysteresis loop. The average pore diameter was calculated to be 9.08 nm, which is larger than the values reported in our previous study.^59^ The BET surface area was found to be 9.38 m^2^ g^–1^. The FESEM imaging revealed a well-defined porous nanosized network. The clear and distinct small pores were visible in the pristine probe material (Figure 10a), which clearly appeared filled with pores after exposure to TNT vapors (Figure 10b), indicating physical adsorption of the analyte into the porous matrix. This porosity and surface area are likely responsible for the effective adsorption and preconcentration of TNT vapors, thereby facilitating their trace-level detection.^54^

**Figure 10:**
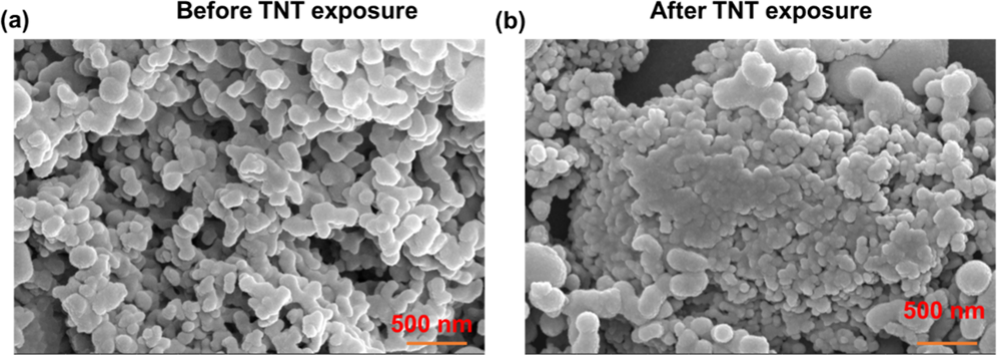
FESEM images of the polymer (a) before and (b) after TNT vapor exposure.

## Conclusion

A donor–acceptor (D–A) based AIEE active porous organic polymer P1 was designed and synthesized (*M̅*_w_, ∼44000 Da). The utilization of a strong accepting unit –SO_2_ in conjugation with an imine through a phenyl spacer was done. The synthesized polymer shows a response toward PA and TNT in the aqueous phase with a detection limit of 1.5 ppm and 5.54 ppm, respectively. The work has been extended to show the paper strip-based sensing of TNT vapor, and a detection limit of 50 ppb was observed. The FESEM imaging of the polymer suggests the presence of porosity, which was further confirmed by a BET surface analysis. The mechanism of quenching was investigated thoroughly using experiments and DFT, and FRET was found to be responsible for emission quenching when PA was introduced, while PET was found to be the main cause when TNT was introduced. The incorporation of strong donor and acceptor moieties into a framework leads to better sensitivity. It helps to lower the LUMO energy level of the polymer, facilitating better possible electron transfer to the LUMO of TNT. Thus, this work may inspire further development of a sensitive porous polymer with a strong donor–acceptor character for more effective detection of various nitro explosives.

## Supplementary Material



## Notes

Additional experimental details, materials, calculations, and instrumentation details, including a photograph of the experimental setup, Lippert plots, and photophysical and sensitivity studies (PDF)
