# Supplementary material for: Enhanced TNT Vapor Detection via a Donor–Acceptor-Based Imine Cross-Conjugated Aggregation-Induced Enhanced Emission Active Porous Polymer
Source: ACS Omega. 2025 Sep 25;10(39):46141–52. doi: 10.1021/acsomega.5c08259 (PMC12508984; doi:10.1021/acsomega.5c08259)
Supplement: Supplementary file 1 [file ao5c08259_si_001.pdf]

Supporting Information

Enhanced TNT Vapor Detection Via Donor-Acceptor Based Imine Cross-Conjugated Aggregation-Induced Enhanced Emission Active Porous Polymer

Pramod C. Raichure,[a][b]^ *Annu Agarwal,* [a]^ *Bharat Kaushik,*[a] *Ajeet Singh,*[a] *and Inamur Rahaman Laskar**[a]

[a] Department of Chemistry, Birla Institute of Technology and Science, Pilani, Pilani Campus, Vidya Vihar, Pilani, Rajasthan 333031, India

[b] Department of Chemistry, Indian Institute of Technology (IIT), Bombay, Mumbai, India 400076

^Pramod C. Raichure and Annu Agarwal have contributed equally.

ir_laskar@pilani.bits-pilani.ac.in

Table of contents

Section 1: Materials, instrumentation, and calculations.

Section 2: Characterisations of Synthesised Compounds

Figure S1: ^{1}H NMR spectra of L2 in $CDCl_3$ solvent.

Figure S2: ^{13}C NMR spectra of L2 in $CDCl_3$ solvent.

Figure S3: ^{1}H NMR spectra of M1 in $CDCl_3$ solvent.

Figure S4: ^{13}C NMR spectra of M1 in $CDCl_3$ solvent.

Figure S5: ^{1}H NMR spectra of P1 in DMSO-d_6 solvent.

Figure S6: ^{13}C NMR spectra of P1 in DMSO-d_6 solvent.

Figure S7: GPC analysis of the chloroform solution of the polymer.

Section 3: Photophysical studies of the synthesised compounds

Figure S8: Excitation-dependent emission spectra of polymer in (a) THF solution, and (c) in the solid state. Contour plots for the same (b) in THF solution, and (d) in the solid state.

Figure S9: Lippert Mataga plot of the polymer in various solvents

Figure S10: Plot of the Dimroth-Reichardt polarity parameter $E_T(30)$ with (a) absorption energy E_a and emission energy E_f, (b) Stokes shift.

Figure S11: Emission property study of the probe recorded in THF and PEG. (a) Emission of 0, 20, 50, 70, and 90% PEG solutions of P1 under UV lamp (λ_{ex} = 365 nm), (b) PL spectra of the solutions excited

at 380 nm.), (b) The corresponding PL spectra of the solutions excited at 380 nm, (c) particle size of P1 recorded in THF solution, and (d) particle size of 90% water: THF AIE solution of P1.

Figure S12: Lifetime decay plot of the 90% water: THF AIE solution of polymer P1.

Figure S13: Linear fit of SV plot of PL titration with analytes. (a) with aqueous PA, (b) with aqueous TNT, and (c) with Vapor TNT.

Figure S14: Lifetime spectra of the 90% water: THF AIE solution of the polymer P1 in the absence and presence of analytes TNT

Figure S15: Filter paper-based detection of the analytes (aqueous solutions of picric acid and TNT) by impregnating the probe with Whatman filter paper (λ_{ex} = 365 nm).

Figure S16: Selectivity test of P1 (λ_{ex} = 365 nm) (a) in common volatile solvents, and (b) in different nitroexplosives

Figure S17: Calculation of HOMO and LUMO energies of the polymer (in eV) P1 using (a) absorption spectra, and (b) cyclic voltammetry plot.

Figure S18: Time-dependent study of the polymer emission without exposure to the analyte (λ_{ex} = 380 nm).

Figure S19: BET plot of the polymer P1 (Powder form).

Figure S20: Experimental setup for TNT Vapor Detection.

Table S1: Details of the solvatochromic study of the polymer P1 in various solvents.

Table S2: Details of the parameters required for the plot of Dimroth-Reichardt polarity parameter $E_T(30)$ with absorption and emission energies, and with the Stokes shift, in various solvents.

Materials

Triphenylamine was purchased from TCI, and phosphorus oxychloride was procured from Spectrochem. 4,4'-sulphonyldianiline was purchased from TCI, and Acetic acid was purchased from Spectrochem. $MgSO_4$ was purchased from Merck. UV-grade solvents and trifluoroacetic acid were purchased from Spectrochem and used without further purification. Triple-deionised water was used throughout the experiment.

Instrumentation

^{1}H NMR and 13C NMR spectra were recorded using a 400 MHz Bruker NMR spectrometer. GPC was recorded on PerkinElmer (model Turbo Matrix-40). UV-VIS absorption spectra were recorded using a Shimadzu Spectrophotometer (models UV-1800 and UV-2550). GPC analysis was done on a Waters Associates Gel Permeation Chromatography system. Polystyrene was used as an internal standard. Steady-state photoluminescence (PL) spectra were recorded on a Horiba Jobin Yvon Spectrofluorometer (FluoroMax-4 and FluoroMax plus) and a Jasco Fluorimeter. The lifetime of the complexes was recorded on Horiba Delta Flex 01. Quantum yield of the polymer was recorded on a Horiba Jobin Yvon Spectrofluorometer, FluoroMax plus. DLS study was performed on Malvern Zeta Sizer. FESEM images were taken on FEI: Apreo LoVac. BET was recorded on a Quantachrome instrument.

Calculations

Solvatochromic studies

Solvatochromic studies were performed in various UV grade solvents. The Lippert-Mataga Plot was plotted according to the equation given below

$\Delta\nu = \frac{2\Delta\mu^2}{hca^3}\Delta f$ + Constant

Where, $\Delta f = \frac{\epsilon-1}{2\epsilon+1} - \frac{\eta^2-1}{2\eta^2+1}$,

a = Onsager cavity

h = Plank's Constant

c = speed of light,

$\Delta\mu$ = Change in dipole moment,

ϵ= dielectric constant of the solvent

η= refractive index of the solution

Values of the Dimroth-Reichardt polarity parameters were taken from the literature.[1]

Calculation of quenching constant

The value of the quenching constant for static quenching can be obtained by using the formula given below,
$F_0/F = 1 + K_{sv}[Q]$

Where F_0 is the probe's initial intensity without the addition of analyte, F is the intensity of the probe after adding the quencher (TNT) of known concentration. K_{sv} is the Stern-Volmer quenching constant, and [Q] is the concentration of quencher (TNT).

For solution-state detection of TNT

$$\frac{F_0}{F} = 1 + K_{SV}[Q]$$

$$= \frac{127798}{47459} = 1 + K_{SV} \times \ 208 \times 10^{-6} M$$

$$\Rightarrow \frac{2.6928 - 1}{208 \times 10^{-6}} = K_{SV}$$

$$K_{SV} = 0.0081 \times \ 10^6 M$$

For Vapor phase detection of TNT,

$$\frac{F_0}{F} = 1 + K_{SV}[Q]$$

$$= \frac{406833}{297580} = 1 + K_{SV} \times \ 127.2 \text{ ppb}$$

$$\Rightarrow \frac{1.367 - 1}{56 \times 10^{-8}} = K_{SV}$$

$$K_{SV} = 0.65 \times \ 10^6 M$$

For picric acid

The value of the apparent quenching constant (K_{app}), where both static and dynamic quenching were present, can be calculated using the formula

$$\frac{F_0}{F} = 1 + K_{app}[Q]$$

$$= \frac{134550}{6536} = 1 + K_{app} \times \ 234 \times 10^{-6} M$$

$$\Rightarrow \frac{20.58 - 1}{234 \times 10^{-6}} = K_{app}$$

$$K_{app} = 0.0837 \times \ 10^6 M$$

Calculation of limit of detection

The LOD was derived by entering the obtained values of k and σ into the equation below.

LOD = 3 σ/k

For solution state detection of TNT

$$\text{LOD} = \frac{3\sigma}{k}$$

$$\mathrm{LOD} = \frac{3 \times 0.069}{0.0085}\,\mu\mathrm{M} = 24.42\ \mu\mathrm{M} = 5.54\ \mathrm{ppm}$$

For Vapor phase detection of TNT

$$\mathrm{LOD} = \frac{3 \times 0.046}{0.0028}\,\mathrm{ppb} = 50\ \mathrm{ppb}$$

For solution state detection of PA

$$\mathrm{LOD} = \frac{3 \times 0.069}{0.031}\,\mu\mathrm{M} = 6.67\ \mu\mathrm{M} = 1.50\ \mathrm{ppm}$$

^{1}H NMR (400 MHz, $CDCl_3$) δ 7.77 (d, J = 8.7 Hz, 4H), 7.40 (dd, J = 8.2, 7.4 Hz, 2H), 7.28 (s, 1H), 7.21 – 7.15 (m, 6H).

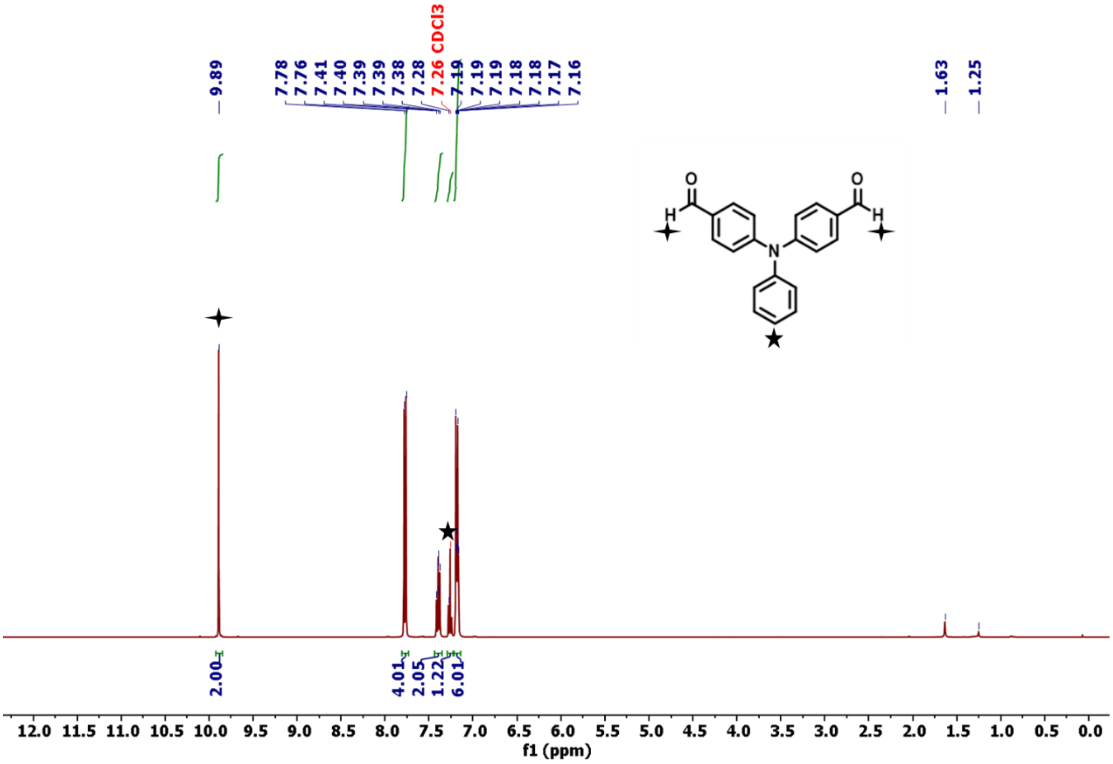

Figure S1: ^{1}H NMR of L2 in $CDCl_3$ solvent.

^{13}C NMR (101 MHz, $CDCl_3$) δ 190.53, 152.03, 145.53, 131.32, 131.31, 130.17, 127.08, 126.28, 122.78.

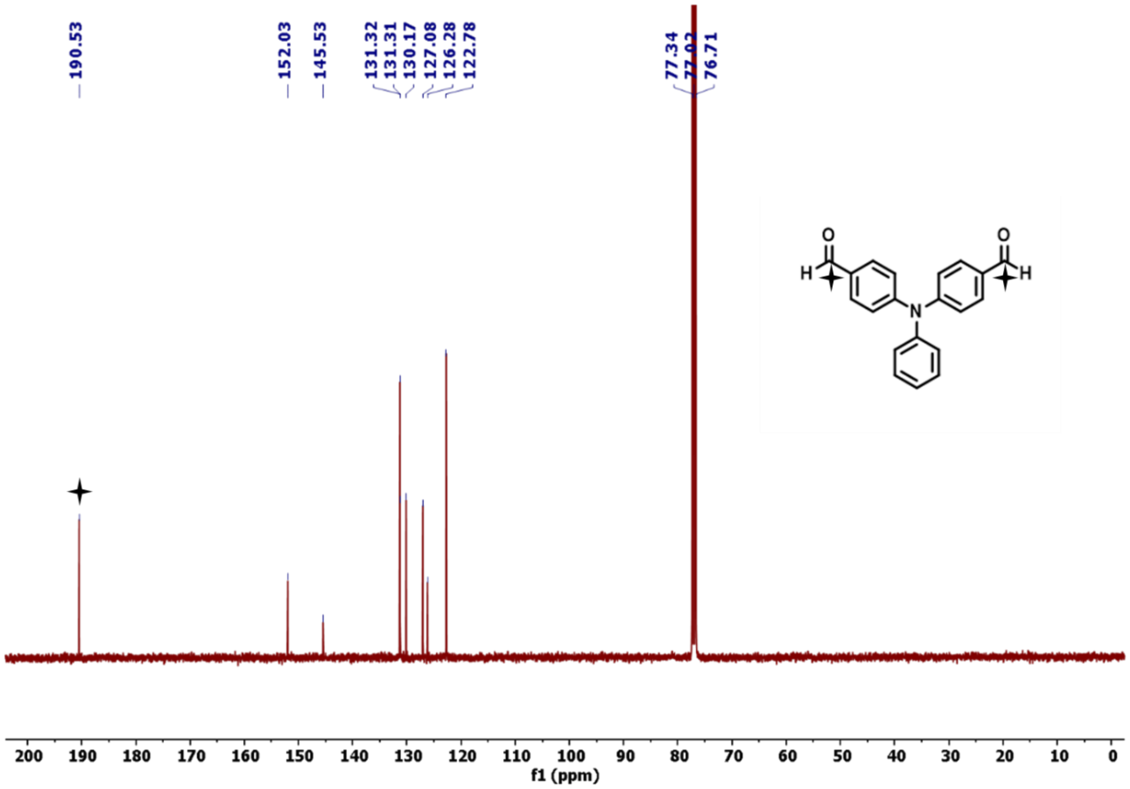

Figure S2: ^{13}C NMR of L2 in $CDCl_3$ solvent.

^{1}H NMR (400 MHz, Chloroform-d) δ 9.95 (s, 3H), 7.84 (d, J = 8.6 Hz, 6H), 7.25 (d, J = 9.3 Hz, 6H).

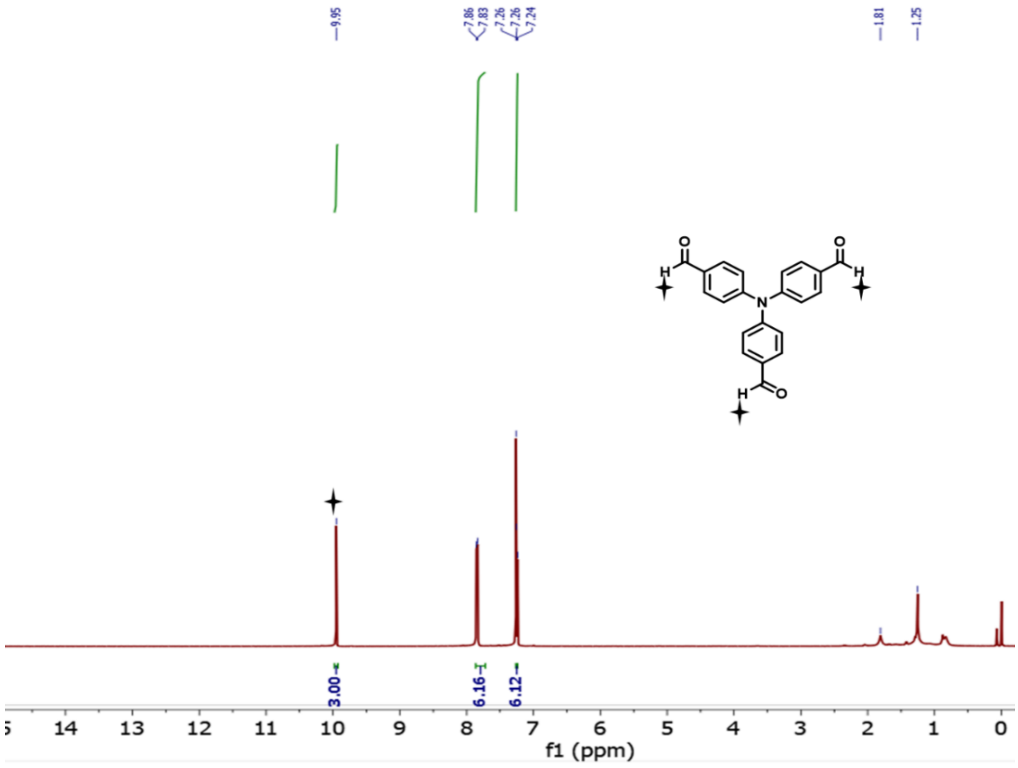

Figure S3: ^{1}H NMR of M1 in $CDCl_3$ solvent.

^{13}C NMR (101 MHz, Chloroform-d) δ 190.43, 151.20, 132.63, 131.49, 124.54.

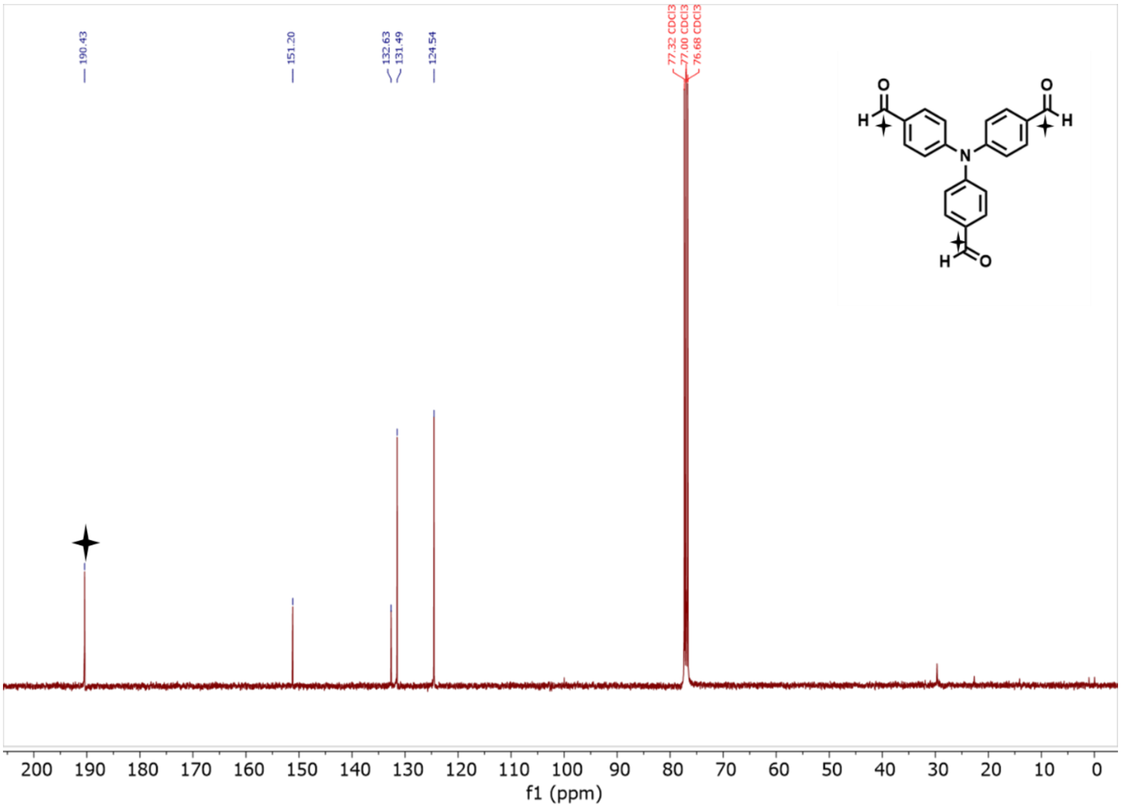

Figure S4: ^{13}C NMR of M1 in $CDCl_3$ solvent.

^{1}H NMR (400 MHz, DMSO-d$_6$)

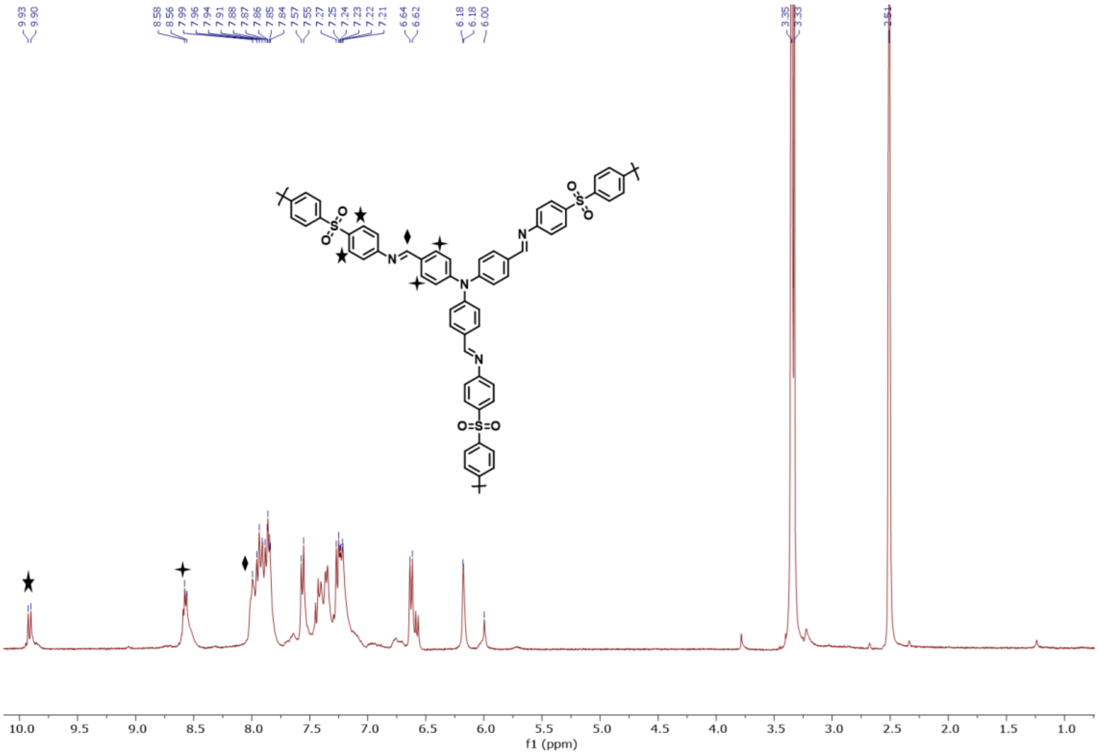

Figure S5: ^{1}H NMR of P1 in DMSO-d$_6$ solvent.

^{13}C NMR (101 MHz, DMSO-d$_6$) δ 192.00, 153.19, 151.23, 132.69, 131.87 (d, J = 12.6 Hz), 128.99, 128.59, 124.92, 123.11, 113.30.

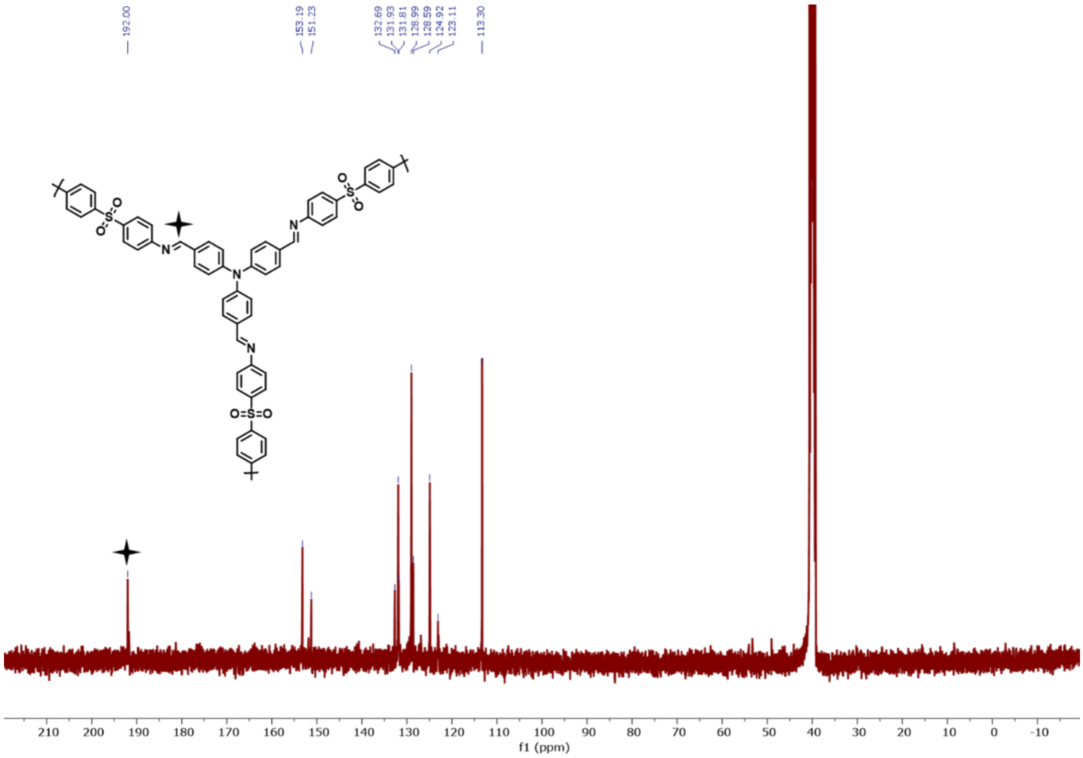

Figure S6: ^{13}C NMR of P1 in DMSO-d$_6$ solvent

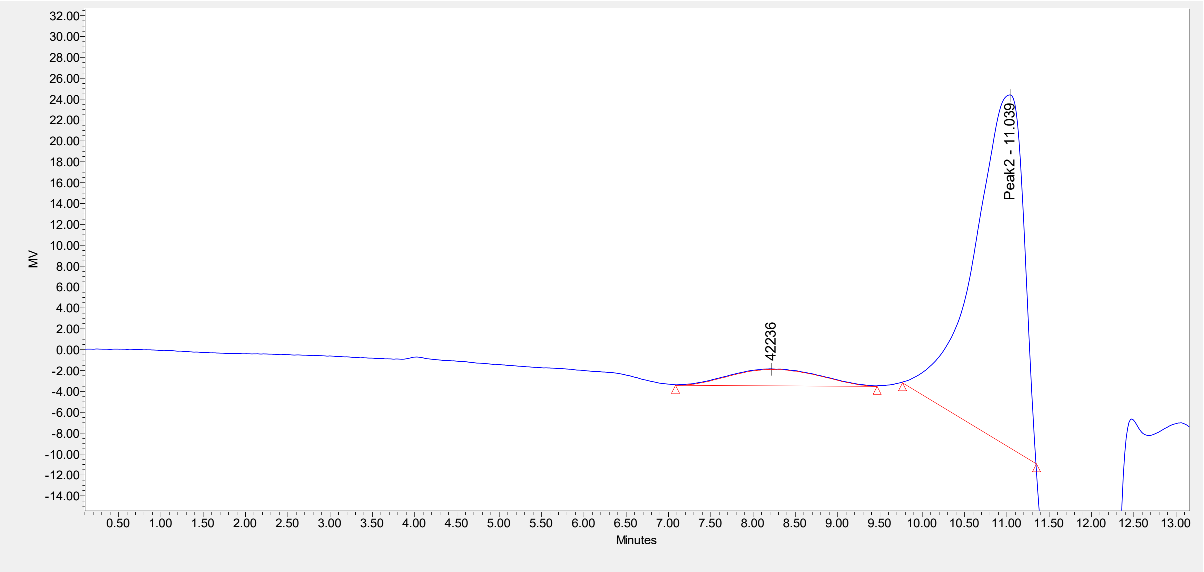

	Name	Retention Time	Mn	Mw	MP	Mz	Mz+1	Polydispersity	Mz/Mw
1	Broad	8.215	34456	44741	42236	56742	68770	1.298499	1.268222
2	Peak2	11.039	2202	2406		2700	3090	1.092659	1.121930

Figure S7: GPC analysis of the chloroform solution of the polymer.

Results and Discussions-

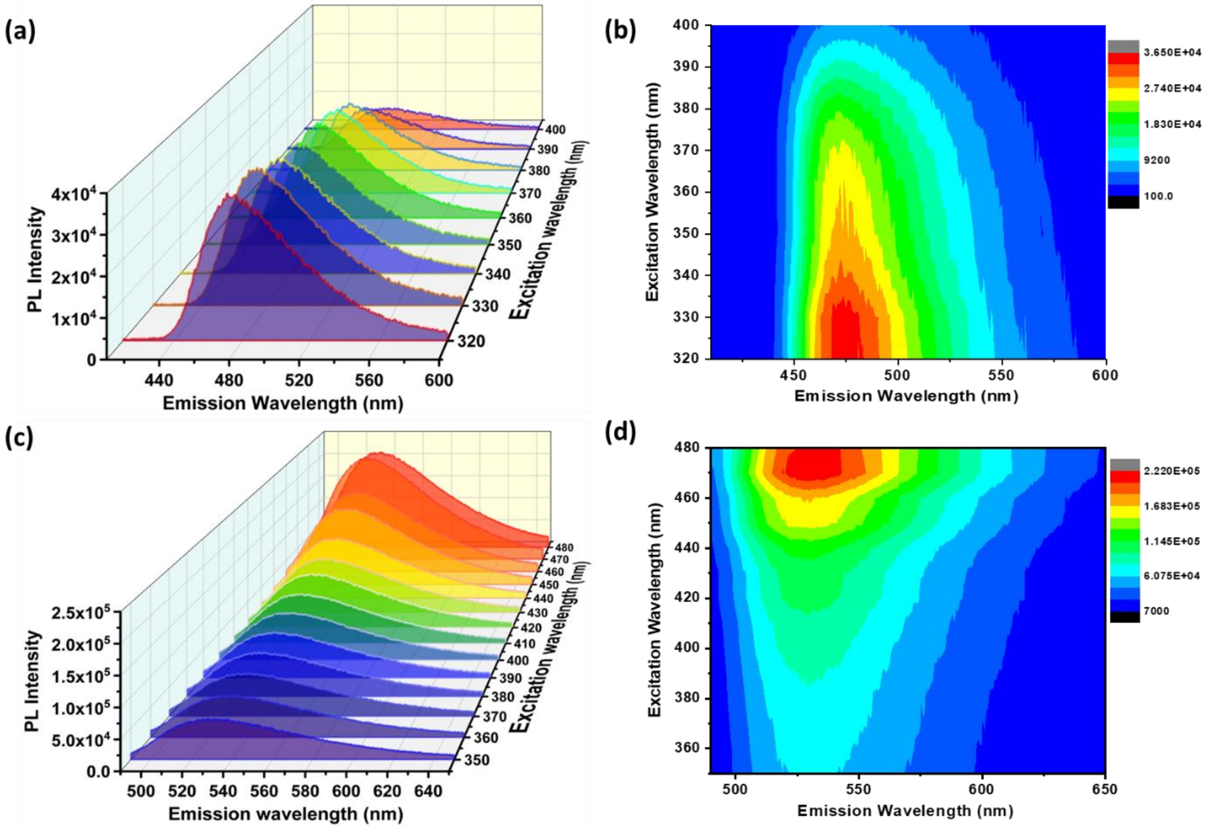

Figure S8: Excitation-dependent emission spectra of polymer in (a) THF solution, and (c) in the solid state. Contour plots for the same (b) in THF solution, and (d) in the solid state.

Table S1: Details of the Solvatochromic study of the polymer P1 in various solvents.

Sr. No.	Solvent	Absorption Maxima (nm)	Emission Maxima (nm)	Stokes Shift (nm)	Polarity Index[2]	Dielectric Constant	Refractive index
**1.**	Toluene	371	427	56	2.4	2.4	1.496
**2.**	DCM	379	460	81	3.1	9.08	1.424
**3.**	THF	375	460	85	4.0	7.60	1.407
**4.**	Acetone	372	470	98	5.1	20.7	1.358
**5.**	DMF	376	472	96	6.4	36.70	1.430
**6.**	DMSO	381	482	101	7.2	46.68	1.479

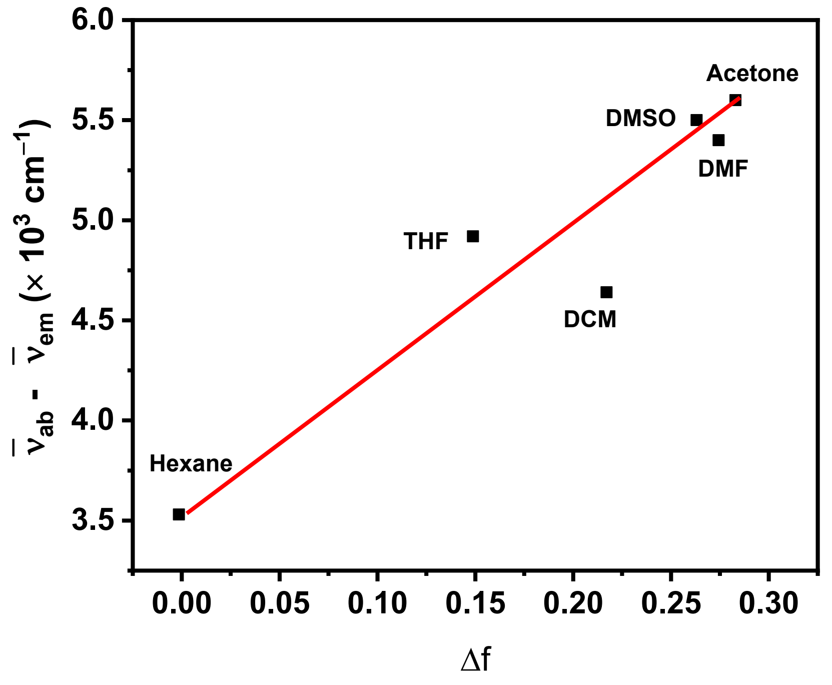

Figure S9: Lippert Mataga plot of the polymer in various solvents.

Table S2: Details of the parameters required for the plot of Dimroth-Reichardt polarity parameter $E_T(30)$ with absorption and emission energies, and with the Stokes shift, in various solvents.

Sr. No.	Solvent	Absorption energy (E_a = 28591/λ_{max})	Emission energy (E_f = 28591/λ_{max})	$E_T(30)$
**1.**	Toluene	77.06	66.95	33.9
**2.**	DCM	75.4	62.15	40.7
**3.**	THF	76.2	62.15	37.4
**4.**	DMSO	75.04	59.32	45.1

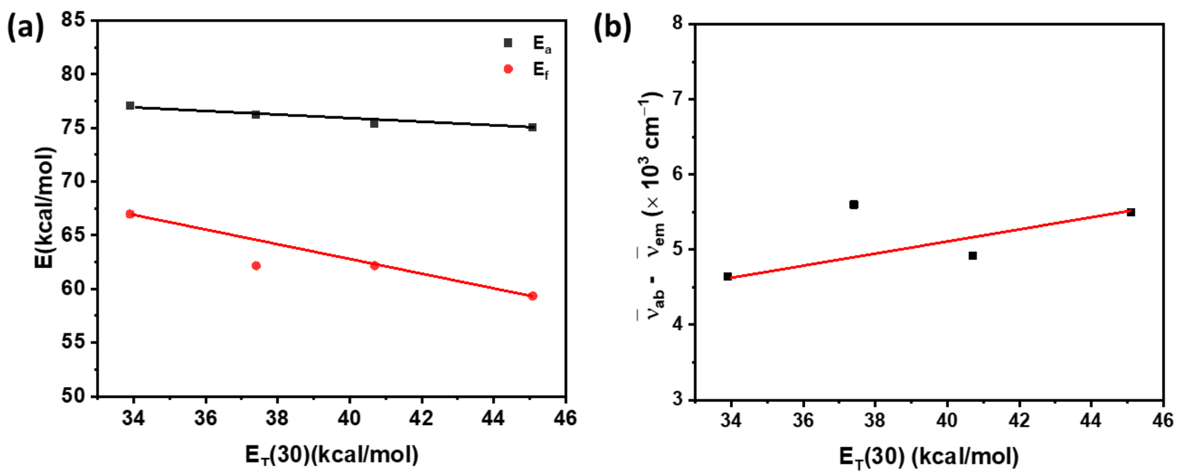

Figure S10: Plot of the Dimroth-Reichardt polarity parameter $E_T(30)$ with (a) absorption energy E_a and emission energy E_f, (b) Stokes shift.

THF-PEG mixture PL studies-

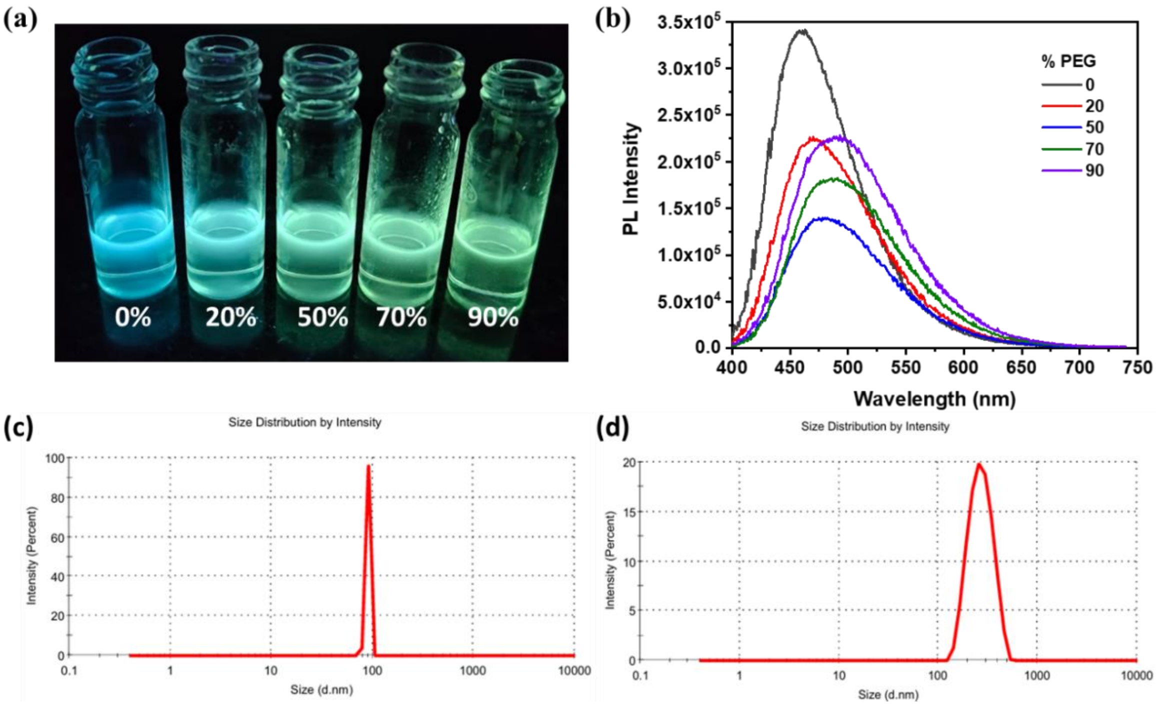

Figure S11: Emission property study of the probe recorded in THF and PEG. (a) Emission of 0, 20, 50, 70, and 90% PEG solutions of P1 under UV lamp (λ_{ex} = 365 nm), (b) The corresponding PL spectra of the solutions excited at 380 nm, (c) particle size of P1 recorded in THF solution, and (d) particle size of 90% water: THF AIE solution of P1.

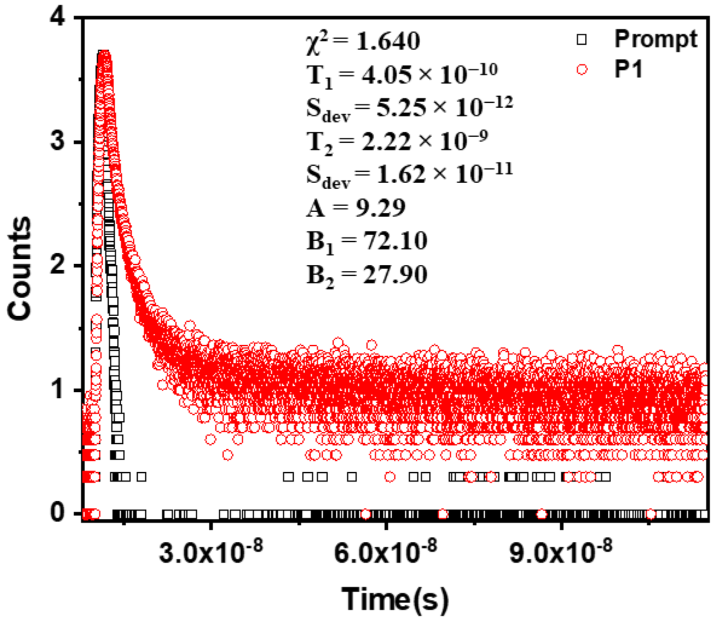

Figure S12: Lifetime decay plot of the 90% water: THF AIE solution of polymer P1.

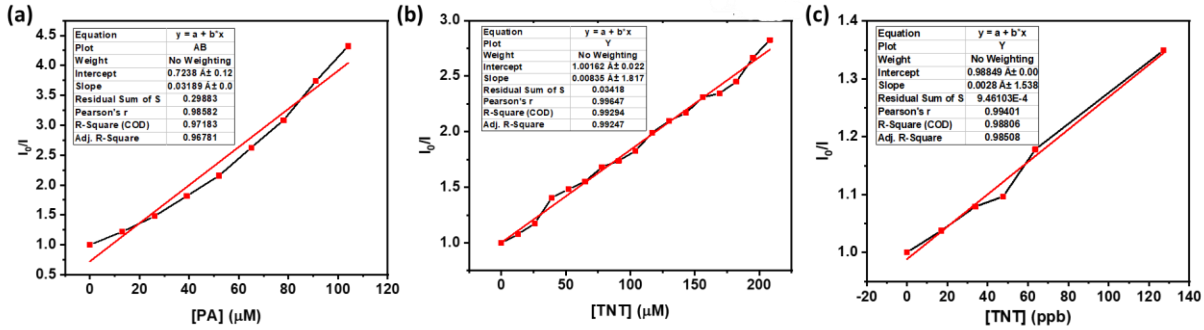

Figure S13: Linear fit of SV plot of PL titration with analytes. (a) with aqueous PA, (b) with aqueous TNT, and (c) with Vapor TNT.

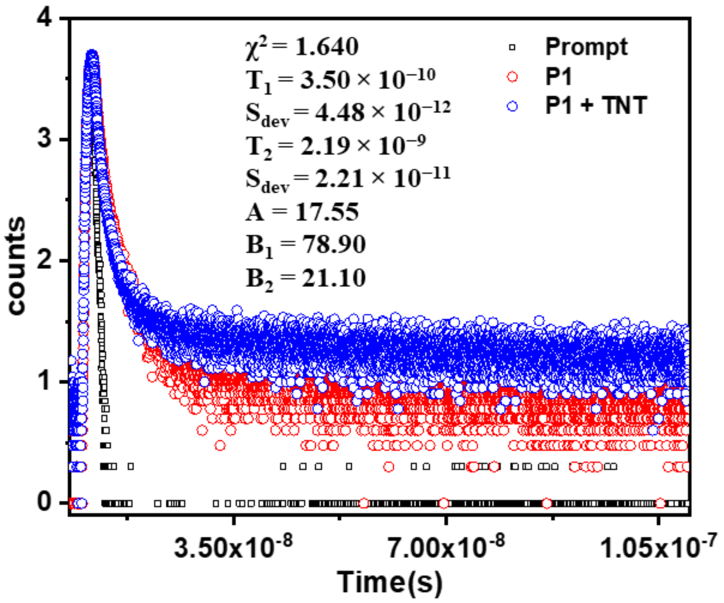

Figure S14: Lifetime spectra of the 90% water: THF AIE solution of the polymer P1 in the absence and presence of the analyte TNT.

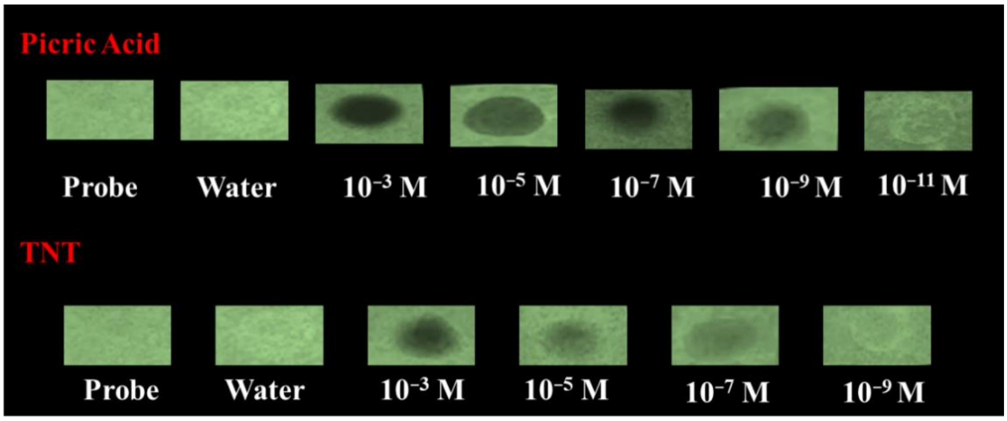

Figure S15: Filter paper-based detection of the analytes (aqueous solutions of picric acid and TNT) by impregnating the probe with Whatman filter paper (λ_{ex} = 365 nm).

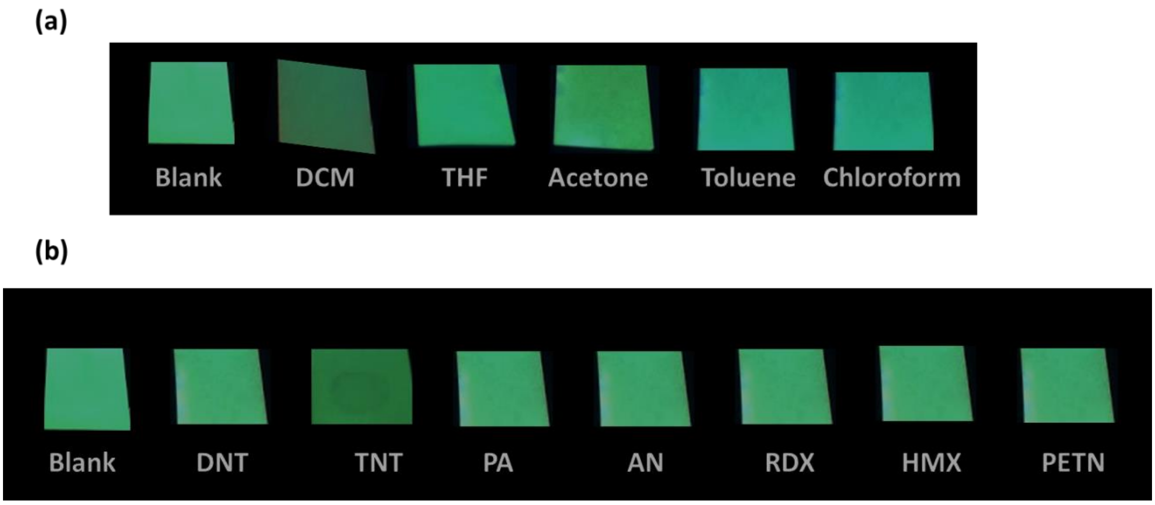

Figure S16: Selectivity test of P1 (λ_{ex} = 365 nm) (a) in common volatile solvents, and (b) in different nitroexplosives.

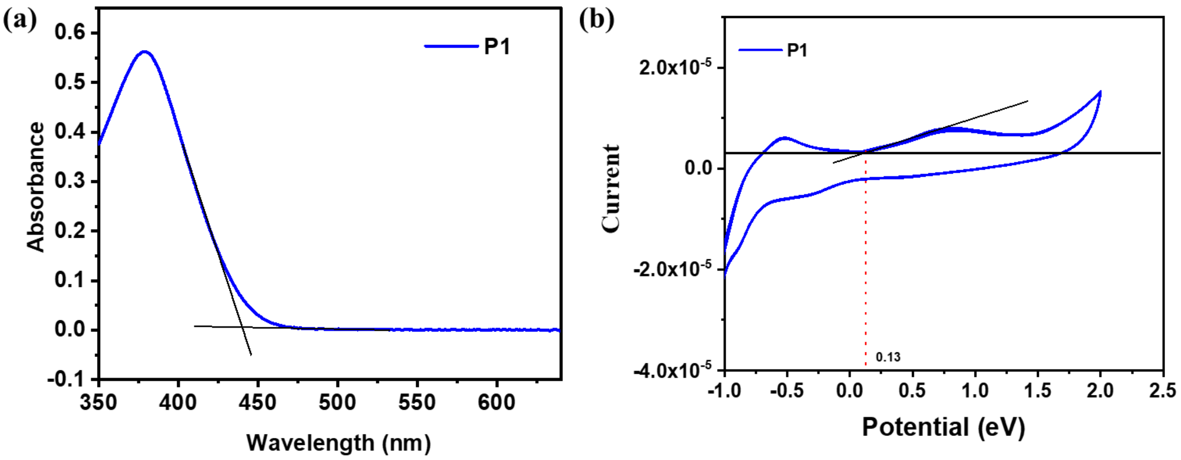

Crosspoint wavelength $= \Delta E = 439\ nm$
$1\ eV = 1240\ nm$
$$\Rightarrow 439\ nm = \frac{1240}{439}\ eV = 2.82\ eV$$

Cross point potential = 0.15 eV
$HOMO = -(0.13 + 4.4)\ eV = -4.53eV$
$LUMO - HOMO = \Delta E$
$\Rightarrow LUMO = \Delta E + HOMO$
$= (2.82 - 4.53)eV = -1.71\ eV$

Figure S17: Calculation of HOMO and LUMO energies of the polymer (in eV) P1 using (a) absorption spectra, and (b) cyclic voltammetry plot.

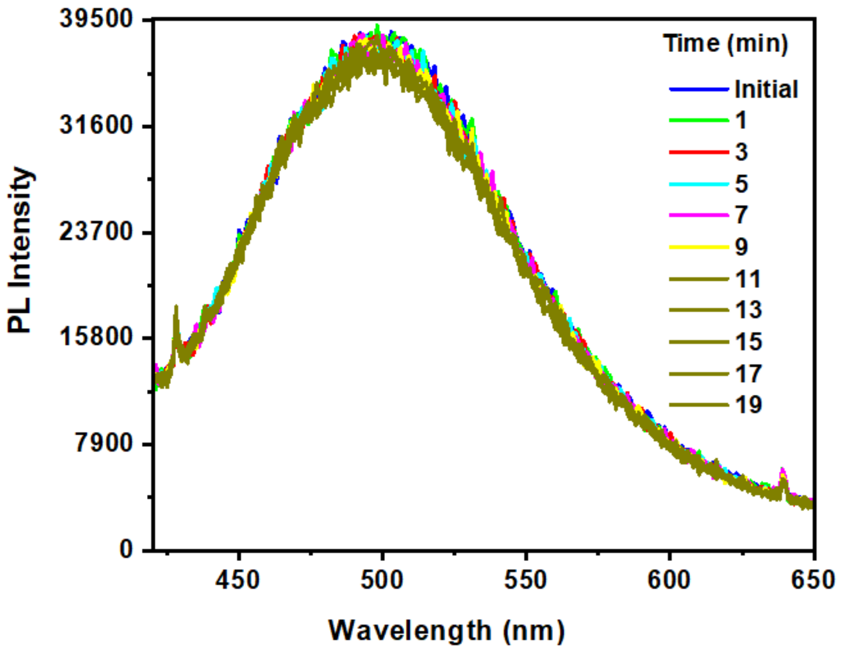

Figure S18: Time-dependent study of the polymer emission without exposure to the analyte (λ_{ex} = 380 nm).

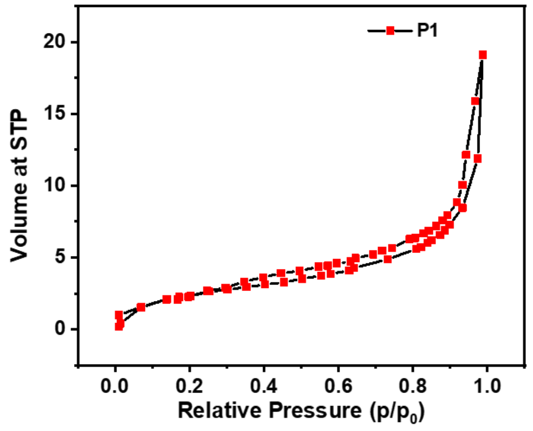

Figure S19: BET plot of the polymer P1 (powder form).

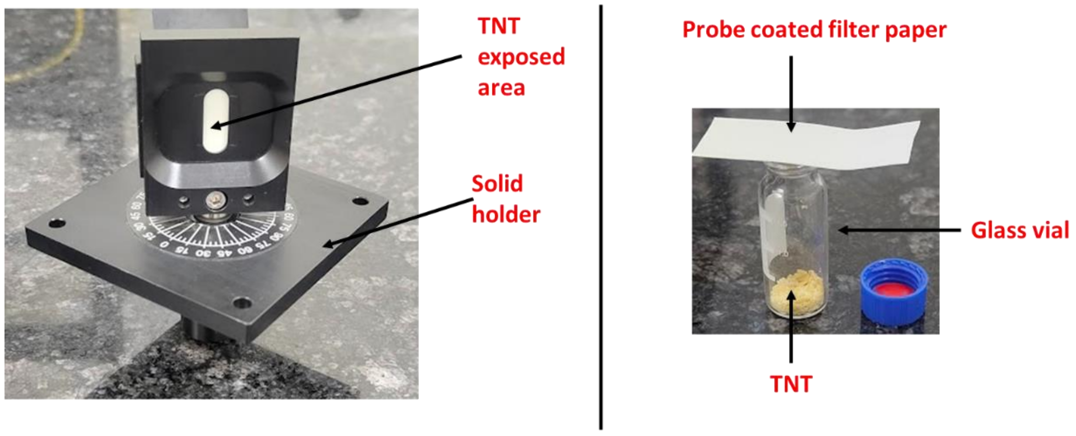

Figure S20: Experimental setup for TNT Vapor detection.

References

1. AbhijnaKrishna, R.; Wu, S.-P.; Velmathi, S., Photophysical Insights and Polarity Dynamics of 4-Diethylamino Salicylaldehyde-Based Fluorescent Molecules and Their Application as Mitochondrial and Endoplasmic Reticulum Tracker Agents. *ACS Applied Optical Materials* **2025,** *3* (6), 1278-1286.
2. Li, C.-P.; Du, M., Role of solvents in coordination supramolecular systems. *Chemical Communications* **2011,** *47* (21), 5958-5972.
